# Tension zone trapped by exogenous cline: Analysis of a narrow hybrid zone between two parapatric *Oxytropis* species (Fabaceae)

**DOI:** 10.1002/ece3.9351

**Published:** 2022-09-20

**Authors:** Hui Wang, Xin‐Nuo Li, Song‐Hua Mo, Min Wang, Pei‐Liang Liu, Qin Li, Zhao‐Yang Chang

**Affiliations:** ^1^ College of Life Sciences Northwest A&F University Yangling Shaanxi China; ^2^ Key Laboratory of Resource Biology and Biotechnology in Western China Northwest University Xi'an Shaanxi China; ^3^ Department of Science and Education Field Museum Chicago Illinois USA

**Keywords:** bimodal, ecotone, intrinsic incompatibility, *Oxytropis*, tension zone

## Abstract

Hybrid zones have been widely highlighted for their interest in understanding evolutionary processes. It is generally accepted that hybrid zones can be maintained in a balance between dispersal and selection. However, the selective forces can either be endogenous (i.e., genetic incompatibilities between parental taxa) or exogenous (i.e., parental taxa are adapted to different environments). In this study, we evaluated these alternatives and determined the maintenance of a narrow hybrid zone between parapatric distributed *Oxytropis diversifolia* and *O. leptophylla* in Nei Mongol, China. For 507 individuals sampled from two populations in the hybrid zone, 12 *O. diversifolia* populations and five *O. leptophylla* populations, we measured leaf‐morphological characteristics, quantified genetic structure using 11 nuclear microsatellite loci and five chloroplast DNA intergenic regions, collected micro‐ and macrohabitat data, and conducted geographical cline analysis. We found that the two species differed in leaf morphology, and putative hybrids showed either intermediacy or a bias to *O. diversifolia*. Parental taxa formed two genetically distinct clusters, while populations in the hybrid zone consisted of both parental forms and various admixed individuals, exhibiting a bimodal pattern. The hybrid zone was coupled to ecological transitions of both microhabitat (i.e., the slope) and macroclimatic conditions. However, the genetic clines were significantly narrower than the environmental cline. Our results indicate that endogenous selection can be primarily responsible for maintaining the hybrid zone, while local adaptation accounts for the position of the zone. We further suggest the probable outcome of hybridization could be introgression.

## Graphical abstract

In this study, we analyzed a narrow hybrid zone generated between two parapatric Oxytropis species in Nei Mongol, China. We found that the genetic clines are abrupt and coincident, while the environmental clines are wide or gradual. Our results indicate that endogenous selection can be primarily responsible for maintaining the hybrid zone, and local adaptation accounts for the position of the zone.

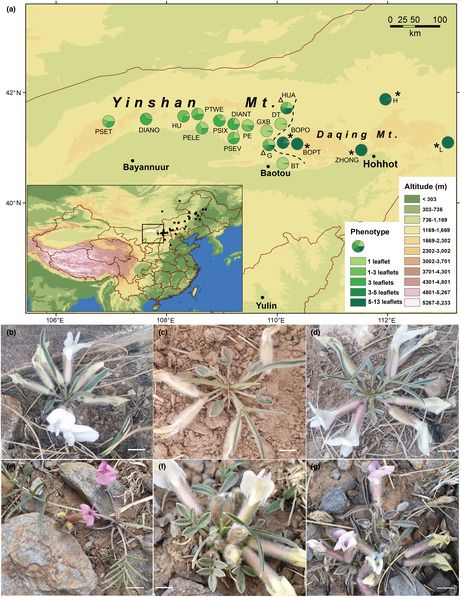

## 1 INTRODUCTION

Natural hybridization, the mating of individuals from distinguishable populations, usually of distinct taxa, frequently occurs in plants and can have diverse evolutionary consequences (Abbott et al., 2013; Buerkle et al., 2003; Harrison, 1993; Hopper, 1995; Whitney et al., 2010). Hybridization may slow or reverse differentiation by allowing gene flow and recombination, and consequently results in breakdown of species boundaries and cause extinction of rare endemics (Buerkle et al., 2003; Levin et al., 1996; Rhymer & Simberloff, 1996; Wolf et al., 2001). On the other hand, hybridization may contribute to speciation through the formation of new hybrid taxa (either via allopolyploidy or homoploid hybrid speciation), and introgression of a few loci may also promote adaptive divergence and so facilitate speciation (reviewed in Abbott et al., 2013). Moreover, divergent lineages with incomplete barriers of reproduction may form stable hybrid zones, which can be examined to determine mechanisms that reduce gene flow and genome merging (Abbott, 2017; Stankowski et al., 2021). Depending on the hybridizing taxa involved, an assessment of the evolutionary outcome of hybridization can provide important insights in predicting species persistence and hybrid speciation, as well as the maintenance of biological diversity (Yan et al., 2017).

Hybrid zones are great “natural laboratories” offering many insights into evolutionary processes (Harrison, 1993; Hewitt, 1988). Most hybrid zones have existed for an extended time, so they integrate the outcome of evolutionary processes over timescales that are not accessible in laboratory experiments (Nolte et al., 2006). Hybrid zones often originate from divergent lineages that are evolving independently, and come into secondary contact at the edges of their distributions. The classic hybrid zone theory (Fisher, 1950; Haldane, 1948) predicts that the extent and shape of hybrid zones depend upon a balance between migration and selection, and the latter often involves a combination of “endogenous” and “exogenous” forms of selection (Barton, 2001; Moore & Price, 1993). Endogenous selection expects that internal genetic incompatibilities reduce the viability or reproductive success of hybrids, and hybrid zones are maintained by a balance between random dispersal and selection against hybrids, which is so‐called “tension zone model” (Barton & Hewitt, 1985). In this model, the antihybridization mechanism would be expected independent of ecological conditions (Swenson, 2008). Exogenous selection assumes that the fitness of genotypes is coupled to the environment, and that selection favors different genotypes in different environments (Barton & Gale, 1993; Moore & Price, 1993). As a result, hybrid zones tend to be located in ecotones between habitats occupied by parental forms (i.e., ecotone model; Nolte et al., 2006). An ecotone model predicts selection against pure genotypes in alternative habitats, and less selection against hybrids if the latter have an intermediate degree of habitat dependent fitness (Nolte et al., 2006).

The hybrid zone literature has long debated the relative roles of endogenous and exogenous selection (Arnold, 1997; Barton, 2001), and in many cases both exogenous and endogenous factors are acting concomitantly (Bierne et al., 2011). One way to evaluate these alternatives is to do geographical cline analysis, comparing the width of genetic cline with that of the environmental cline (e.g., Stankowski, 2013; Yanchukov et al., 2006). Furthermore, theoretical modeling and literature review suggest that tension zones have a tendency to become trapped by, and therefore coincide with, exogenous barriers due to ecological selection (Bierne et al., 2011). The result is that local adaptation explains where genetic breaks are positioned, but not necessarily their existence, which can be best explained by endogenous incompatibilities (Bierne et al., 2011). Additional insight can be gained by taking a multilocus approach and examining the frequency distribution of genotypic classes within a hybrid zone. Where a hybrid zone is maintained by weak to moderate postzygotic selection rather than prezygotic barriers, a unimodal distribution is predicted; where postzygotic selection is strong, or prezygotic barriers are present, the distribution is expected to become flatter and, ultimately, bimodal (Jiggins & Mallet, 2000).

It is generally accepted that closely related species tend to hybridize more often. In particular, species in rapidly diversifying adaptive radiations may be particularly prone to hybridization (Abbott et al., 2013; Gourbière & Mallet, 2010; Seehausen, 2004). The genus *Oxytropis* DC. (Fabales: Fabaceae) is recognized to have a history of recent rapid radiation (Kholina et al., 2016; Shahi Shavvon et al., 2017). *Oxytropis* is comprised of about 310 species mainly distributed in temperate regions of the Northern Hemisphere (Zhu et al., 2010), among which the vast majority are restricted to Central and East Asia (Zhao & Liu, 1997). Using nuclear (ITS) and plastid (*trnL*‐*F*) sequence data, Shahi Shavvon et al. (2017) found that *Oxytropis* splits from the close relative *Astragalus* at ca. 15.6 million years ago in the mid Miocene, and the crown group of *Oxytropis* was estimated to date back to 5.6 Ma in the late Miocene. A recent study, which employed the chloroplast genome sequences based on the genome skimming approach, revealed that *Oxytropis* is sister to the Coluteoid clade (*Astragalus* is sister to the *Oxytropis* + Coluteoid clade), and the divergence of *Oxytropis* from the Coluteoid clade was dated to 14.08 Ma (Su et al., 2021). It is suggested that hybridization might have played a significant role in the evolution of *Oxytropis* (Kholina et al., 2016; Malyshev, 2008; Shahi Shavvon et al., 2017). However, the evidence of natural hybridization is comparatively rare, and is based on either morphology or plastid sequence data (e.g., Kholina et al., 2019, 2021). The consequence of natural hybridization has not been explored yet.

**FIGURE 1:**
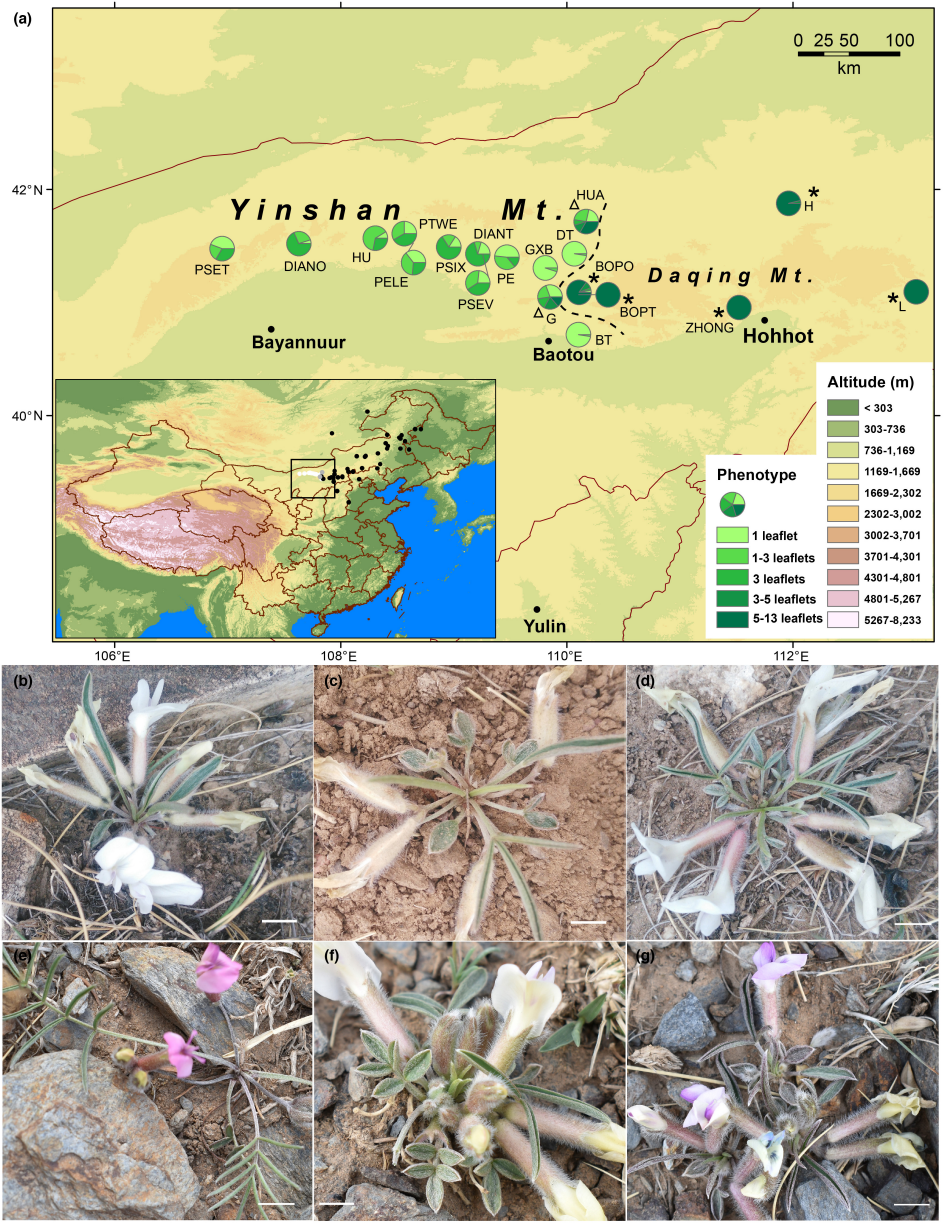
(a) Map of northern China showing locations of 19 sampled *Oxytropis* populations. Each pie chart indicates the proportions of different leaf‐shape phenotypes in the population. Twelve populations are *O. diversifolia*; Δ two populations in the hybrid zone (G and HUA); *five populations of *O. leptophylla*. The dashed line is the contour where the alternative cpDNA haplotypes are predicted to be at a frequency of 0.5. The small inset shows occurrence records of two parental species and the hybrid zone. See section 2.7.2 for details. White circles, *O. diversifolia*; black circles, *O. leptophylla*; gray circles, two populations in the hybrid zone. (b–g) phenotypes of *Oxytropis diversifolia* (a, 1 leaflet; b, 1–3 leaflets; c, 3 leaflets. Flower color: Pale yellow), *O. leptophylla* (d, 5–13 leaflets. Flower color: Purplish red) and putative hybrids (e, 3–5 leaflets with pale yellow flowers; f, 1–3 leaflets with intermediate flower color). Scale bars = 5 mm.

In this study, we analyze a secondary contact between parapatric *Oxytropis diversifolia* E. Peter (Fabales: Fabaceae) and *O. leptophylla* (Pall.) DC. in Nei Mongol, China. Both species belong to sect. *Xerobia*, which is comprised of 27 species (Zhu et al., 2010) that are mostly cryoxerophytes associated with mountain‐steppe territories (Kholina et al., 2021). *O. diversifolia* is mainly distributed in desert‐steppe regions of Nei Mongol, whereas *O. leptophylla* can be found in typical‐steppe and forest‐steppe regions, and is more widely distributed in northeastern China (Nei Mongol, Jilin, Hebei, Shanxi), Mongolia and Russia (Zhao & Liu, 1997; Zhu et al., 2010; Figure 1a). *O. diversifolia* is polymorphic for leaf shape: individuals can have leaves with only 1 leaflet (Figure 1b), 1 to 3 leaflets (Figure 1c), or 3 leaflets (Figure 1d). Populations of this species exhibits clinal variation, where eastern populations are composed chiefly of 1‐leaflet phenotype, and western populations are highly polymorphic with phenotypes of 1–3 leaflets and 3 leaflets more frequent (Figure 1a). The clinal pattern of the heritable leaf‐shape variation can be attribute to natural selection imposed by spatially varying macroclimatic factors, other than to neutral genetic structure (Wang et al., 2021). Morphologically, *O. leptophylla* is distinct from *O. diversifolia* in several characteristics (Figure 1e; Table 1), and the main differences include imparipinnate leaves with 5–13 leaflets and a purple corolla. The distributions of these two species overlap close to the longitude of 110° E, where we found two populations (Figure 1a) in which individuals with intermediate morphology occur (e.g., Figure 1f,g; for details of the morphological differences, see Table 1). It promoted us using molecular markers to verify whether these peculiar individuals represent the offspring of natural hybridization between these species, and if so, to explore what the consequence of natural hybridization is. Furthermore, the hybrid zone is located in ecotones between desert‐steppe regions of *O. diversifolia* and typical‐steppe regions of *O. leptophylla*, and therefore we would like to test whether exogenous selection (i.e., microhabitat and/or macrohabitat factors) against dispersing individuals contribute to shaping and maintaining the hybrid zone.

**TABLE 1:** Differences of descriptive morphological traits between *Oxytropis diversifolia*, *O. leptophylla*, and their putative hybrids

Trait	*O. diversifolia*	Putative hybrids^a^	*O. leptophylla*
Leaves	1 leaflet, 1–3 leaflets, 3 leaflets	1 leaflet, 1–3 leaflets, 3 leaflets, 3–5 leaflets, 5–13 leaflets; sometimes with irregular morphology	5–13 leaflets
Dorsal leaf surface indumentum	Sparse to dense	Glabrous, sparse to less dense	Glabrous
Racemes	1‐ or 2‐flowered	1–5‐flowered	2–5‐flowered
Peduncles	Much shorter than petioles	Much shorter than to as long as petioles	Slightly shorter than to as long as petioles
Corolla color	Pale yellow	Pale yellow, pale yellow with purplish red or blue spots; purplish red	Purplish red, rarely blue

^a^ Putative hybrids from two populations in the hybrid zone were identified based on nuclear microsatellite data, and may include *F*_1_/*F*_2_ hybrids, backcross types and various segregation products.

Here, we use an integrative approach by combining morphological measurements, molecular data, and environmental association analyses to evaluate the pattern and maintenance of the hybrid zone between *O. diversifolia* and *O. leptophylla*. Specifically, we seek to address the following questions: (1) Considering leaf‐morphological traits, what is the phenotypic difference between parental species and putative hybrids? (2) What is the genetic structure of the hybrid zone? Is it unimodal or bimodal? (3) Do micro−/macrohabitat transition showed either coincidence and/or concordance with the genetic clines?

## 2 MATERIALS AND METHODS

### 2.1 Study system

*Oxytropis diversifolia* E. Peter (Fabales: Fabaceae) is a diploid perennial herb occurring at an elevation from 1000 to 2200 m (Zhu et al., 2010). *O. leptophylla* (Pall.) DC. is also a diploid perennial herb occurring at a similar elevation ranging from 800 to 1900 m (Zhu et al., 2010). Sequencing of five cpDNA fragments suggests that *O. leptophylla* and *O. diversifolia* are non‐sister species, with the latter sister to 1‐leaflet *O. neimonggolica* C. W. Chang & Y. Z. Zhao (Wang et al., 2021). *O. leptophylla* and *O. diversifolia* have the same number of chromosomes (2n = 16) (Zhang et al., 1994). *O. diversifolia* is flowering from April to May, while the flowering of *O. leptophylla* occurs slightly later from May to June (Zhu et al., 2010; H. Wang, personal observation). Because the two species and putative hybrids in the hybrid zone have quite similar floral structure (papilionaceous corolla) and floral size (Zhu et al., 2010; H. Wang, personal observation), we mainly measured leaf‐morphological traits in this study.

### 2.2 Population survey and sampling

A total of 19 populations were surveyed in April and May from 2016 to 2018 (Figure 1a; Table S1). Based on leaf‐shape phenotype (i.e., the number of leaflets) and flower color, localities correspond to putatively “pure” populations of *O. diversifolia* (*N* = 12, covering its entire distribution range in Nei Mongol), *O. leptophylla* (*N* = 5, representatives in eastern Nei Mongol), or of the hybrid zone with signs of intermediacy (*N* = 2). In each population, leaf‐phenotype survey was performed by tallying leaf‐phenotype counts (≤100 individuals), or along three to five transects (3 m wide) spanning the length of the population (>100 individuals). This survey was used to estimate population size (i.e., the total number of individuals) and population density (population size divided by total area). Silica‐gel dried leaf samples were collected, using methods described in Wang et al. (2021). In total, 507 individuals were collected, of which 321 were from *O. diversifolia* populations, 102 were from *O. leptophylla* populations, and 84 were from the hybrid zone.

### 2.3 Measurements and analyses of leaf morphology

First, six leaf‐morphological traits were measured by digital caliper for all 507 individuals: early leaf length, early leaf width, mature leaf length, mature leaf width, petiole length of early leaf, petiole length of mature leaf. Second, we quantified the trichome density on leaf upper surface with an Olympus SZ61 stereo microscope (Olympus, Tokyo, Japan). The number of trichomes on a 1 mm × 1 mm surface was counted for five (populations dominated by one phenotype) to 14 individuals (polymorphic populations, maximizing equal sampling for each phenotype) in each parental population. In the hybrid zone, 33 and 28 individuals were quantified at localities G and HUA, respectively. In total, we quantified 211 individuals of all the 19 populations.

Because the six leaf morphological traits were correlated with one other, principal component analysis (PCA) was performed. Data were scaled prior to analyses. The variation in six leaf‐morphological traits, the PCA components, and the trichome density were also analyzed separately by performing linear mixed‐effects models, with category (*O. diversifolia*, *O. leptophylla*, and putative hybrids) as a fixed effect and population nested within category as a random factor.

### 2.4 DNA extraction, nuclear microsatellite genotyping, and chloroplast DNA sequencing

Genomic DNA was extracted from leaf tissue using the cetyltrimethyl ammonium bromide (CTAB) method (Doyle & Doyle, 1990). For the two populations in the hybrid zone, 11 nuclear microsatellite loci (N745892, N145635, N2724893, N2717495, N178451, N161850, N49251, N350553, N935993, N2528349, and N2697375; Table S2) were scored across all 84 individuals according to the methods described by Wang et al. (2018). Five cpDNA intergenic regions (*trn*T‐*psb*D, *pet*N‐*psb*M, *trn*S‐*trn*G, *psb*E‐*pet*L, and *rpl*16 intron; Table S3) were screened for 43 and 21 individuals in G and HUA, respectively, according to the methods described by Wang et al. (2021). All of the sequences were deposited in GenBank under accession numbers MW511796 – MW512115. For the 12 *O. diversifolia* populations and five *O. leptophylla* populations, data of 11 microsatellite genotyping and five cpDNA intergenic‐region sequencing were retrieved from Wang et al. (2021).

### 2.5 Nuclear microsatellite analyses

#### 2.5.1 Population genetics

GENEPOP 4.7 (Rousset, 2008) was used to test for genotypic linkage disequilibrium between each pair of loci within each population, and the probability was adjusted by Benjamini–Hochberg correction (Benjamini & Hochberg, 1995). An assessment of departure from Hardy–Weinberg equilibrium was conducted using exact tests (Rousset & Raymond, 1995) implemented by GENEPOP. For each population, mean allele number per locus (*N*_a_), observed heterozygosity (*H*_o_), unbiased expected heterozygosity (*H*_e_) and *F*_IS_, were calculated using GenAlEx version 6.503 (Peakall & Smouse, 2006, 2012). Those genetic diversity parameters were compared to explore whether they differ between populations located outside and within the hybrid zone.

#### 2.5.2 Parent and hybrid assignment

To estimate the extent of admixture between parental species in the hybrid zone, the Bayesian clustering program STRUCTURE 2.3.4 (Pritchard et al., 2000) was used to assign all 507 individuals. An admixture model with independent allele frequencies were applied, and analyses were conducted with a *K* value that varied from 1 to 10. For each *K* value, analysis was repeated 10 times with a burn‐in of 10,000 Markov Chain Monte Carlo (MCMC) steps followed by 50,000 iterations. The best‐fit number of distinct groups (*K*) was determined based on the maximum Δ*K* value (Evanno et al., 2005) using CLUMPAK (Kopelman et al., 2015). *Tq* is a threshold value used to determine which class an individual should belong to (De hert et al., 2012). *Tq* values previously used to assign parental and hybrid individuals have varied from 0.80 to 0.95 (Yan et al., 2017). In our STRUCTURE analysis, a relatively strict threshold *Tq* value of 0.90 was used, above which the majority of individuals from parental populations were correctly assigned (Table S4). The *Q* scores of individuals in the hybrid zone were then binned in 0.10 increments and compiled in a histogram. The shape of the frequency distribution (either unimodal or non‐unimodal) was determined by Hartigan's dip test (Hartigan, 1985; Hartigan & Hartigan, 1985).

To check the assignment of all individuals to parents (*P*_1_, *P*_2_) and hybrids, two additional methods were used. First, the hybrid index for all individuals were calculated using the *est.h* function from the R package INTROGRESS (Gompert & Buerkle, 2010). Because the parental populations of *O. diversifolia* and *O. leptophylla* did not exhibit fixed differences (i.e., they shared alleles), they were defined as priori to estimate parental allele frequencies. Individuals with hybrid indexes ≤0.20 were assigned to *O. diversifolia*, between 0.20 and 0.80 were assigned to hybrids, and ≥0.80 were assigned to *O. leptophylla*. Second, NEWHYBRIDS Version 1.1 beta (Anderson & Thompson, 2002) was used to compute the posterior probability that individuals fall into six different purebred and hybrid categories. The prior information of the parental populations was given (z0 = *O. diversifolia* and z1 = *O. leptophylla*). Additional parameter settings included a burn‐in of 10,000 MCMC steps followed by 100,000 iterations. A relatively strict posterior probability of 0.95 was used. Results of those two analyses were quite similar to STRUCTURE results, but discordance still existed (Table S4). The final assignments were retrieved with the criteria that two out of the above three methods yielded consistent results.

### 2.6 Chloroplast DNA sequence analyses

Geneious 9.0.2 (http://www.geneious.com) was used to edit, align, and concatenate sequences. Haplotypes were identified using DnaSP v6.12.01 (Rozas et al., 2017), with sites of nucleotide substitutions considered only. With the haplotypes of individuals in hybrid zone localities G and HUA available, GENEPOP was used to test for cytonuclear disequilibrium. Because within‐species diversity is not relevant to the analysis of disequilibria within the hybrid zone (Barton, 2000; Gay et al., 2008), cpDNA haplotypes were set as biallelic (*O. diversifolia* haplotype and *O. leptophylla* haplotype), and multilocus nuclear assignments were used (*O. diversifolia*, putative hybrids, *O. leptophylla*). Moreover, several genetic diversity parameters were calculated for each population using DnaSP, including number of segregating sites (*S*), number of haplotypes (*h*), haplotype diversity (*Hd*) and nucleotide diversity (*π*) (Nei, 1987). A statistical parsimony network based on Median Joining method (Bandelt et al., 1999) was constructed and visualized using PopArt 1.7 (Leigh & Bryant, 2015).

### 2.7 Micro‐ and macrohabitat analyses

#### 2.7.1 Microhabitat differentiation

The microhabitat axes captured habitat attributes at the local scale of individuals within populations. For each individual sampled, a 40 cm × 40 cm square plot was surveyed (Figure S1), slope (by geologic compass), the percent of total vegetation cover, the percent of rocky ground (rock diameter >0.2 cm), and the percent of bare ground (sand and soil) were measured as described in Wang et al. (2021). In total, microhabitat data for 477 individuals in 18 populations were collected (excluding population PSET). Linear mixed‐effects models were conducted on the four variables as described in the “Measurements and Analyses of Leaf Morphology” section.

#### 2.7.2 Macrohabitat differentiation

The macrohabitat axes of bioclimatic data were retrieved based on range‐wide occurrences at the coarse scale. In addition to our field sampling localities (19 records, Table S1), occurrence records of the two parental species were collected from the Chinese Virtual Herbarium (CVH, http://www.cvh.ac.cn), the National Specimen Information Infrastructure (NSII, http://www.nsii.org.cn), and the Global Biodiversity Information Facility (GBIF, http://www.gbif.org) (all accessed August 2019). After removing wrong identifications, duplicate records and observations, and records without georeferences, in total we got 35 additional records for *O. leptophylla*. Nineteen contemporary bioclimatic variables of the time period 1970–2000 at 30‐second resolution were downloaded from WorldClim (http://www.worldclim.org, Fick & Hijmans, 2017). To detect whether these categories (*O. diversifolia*, *O. leptophylla* and the hybrid zone) diverge in present climate conditions, first, one‐way ANOVA on each bioclimatic variable was performed. Among those significantly differentiated variables, three biologically important variables with pairwise Pearson correlation coefficients below 0.7 were then used to conduct PCA, with the entire environmental space built on variable values associated with 10,000 random points sampled from the whole study area.

Moreover, soil characteristics can also be important. For example, in the European Alps, hybrids between the acid‐loving species *Rhododendron ferrugineum* and the basic soil species *R. hirsutum* occur on soils of intermediate pH (Milne & Abbott, 2008). Therefore, soil characteristics of all the 54 localities, including soil pH and FAO (Food and Agriculture Organization) soil units, were retrieved from National Soil Information Service Platform of China (http://www.soilinfo.cn). The associated plant species that co‐occurring at the 19 localities we surveyed were also noted.

### 2.8 Geographical cline analysis

To explore whether endogenous or exogenous selection is primarily responsible for maintaining the hybrid zone, one‐dimensional (1D) cline analysis was performed based on both genetic data (frequency of *O. leptophylla* cpDNA haplotype and microsatellite *Q*_*K2*_ scores) and environmental data (log‐transformed slope values and macroclimatic PC2 scores). Morphological data (leaf‐shape frequency and leaf morphology PC2 scores) were also used to fit geographical cline to test for any effect of divergent selection or morphological introgression.

First, the two‐dimensional sampling locations were collapsed onto a 1D transect according to the method described in Stankowski et al. (2016). Briefly, the frequency of *O. leptophylla* cpDNA haplotype was applied to generate the prediction surface in ARCMAP *v*. 10.2 (Esri), and the position of the cline center in two dimensions were determined (the linear contour in Figure 1a). 1D coordinates for each of the focal populations were obtained by calculating their minimum straight‐line distance from the two‐dimensional cline center, with sites to the west having negative 1D distance values and sites to the east having positive values. Second, the program HZAR (hybrid zone analysis for R, Derryberry et al., 2014) was used to fit all sigmoidal clines. Three‐model comparison were conducted to fit either allele frequency data or quantitative trait data (for details of these models see Brumfield et al., 2001), and the best‐fit model with the lowest Akaike information criterion (AIC) value was selected to estimate cline center and width. Third, the program Cfit7 (Gay et al., 2008) was used to test for cline coincidence and concordance. This program allows two kinds of models compared: one in which different clines constrained to a common center and/or width, and one in which the cline shapes were allowed to vary independently. The constrained model was treated as significantly better than the others using AIC (i.e., the convention of selecting one that has an AIC value smaller by more than two AIC points).

All of the statistical analyses were performed using R software version 3.5.2 (R Core Team, 2018). PCA was performed using the “FactoMineR” library (Lê et al., 2008). Linear mixed‐effects models were fitted using the “lmer” function from the “lme4” library (Bates et al., 2015). Hartigan's dip test was performed using the “DIPTEST” library (Maechler, 2021).

## 3 RESULTS

### 3.1 Spatial pattern of leaf shape distribution

*O. diversifolia* populations showed a significant longitudinal cline (Figure 1a; Table S1): the proportion of 1 leaflet increased from west to east (Spearman's rank test, *r* = 0.70, *N* = 12, *p* = .015), while the proportions of 1–3 leaflets and 3 leaflets exhibited the reverse trend (*r* = −0.56, *p* = .063 and *r* = −0.63, *p* = .029, respectively). We occasionally found two individuals with 3–5 leaflets (one in the population DIANO and one in PE), but they were rare. The five populations of *O. leptophylla* were predominantly monomorphic with the phenotype of 5–13 leaflets, and exceptions were found in BOPO (one with 1 leaflet, one with 3 leaflets, and three with 3–5 leaflets; 15.1%) and H (one with 3–5 leaflets; 4.0%) (Figure 1a; Table S1). In the hybrid zone, five categories of leaf shape coexisted at both localities G and HUA, with proportions of 30.5%/21.4% for 1 leaflet, 22.0%/26.2% for 1–3 leaflets, 14.6%/7.2% for 3 leaflets, 19.5%/11.9% for 3–5 leaflets, and 13.4%/33.3% for 5–13 leaflets, respectively (Figure 1a; Table S1).

### 3.2 Leaf‐morphological variation

**FIGURE 2:**
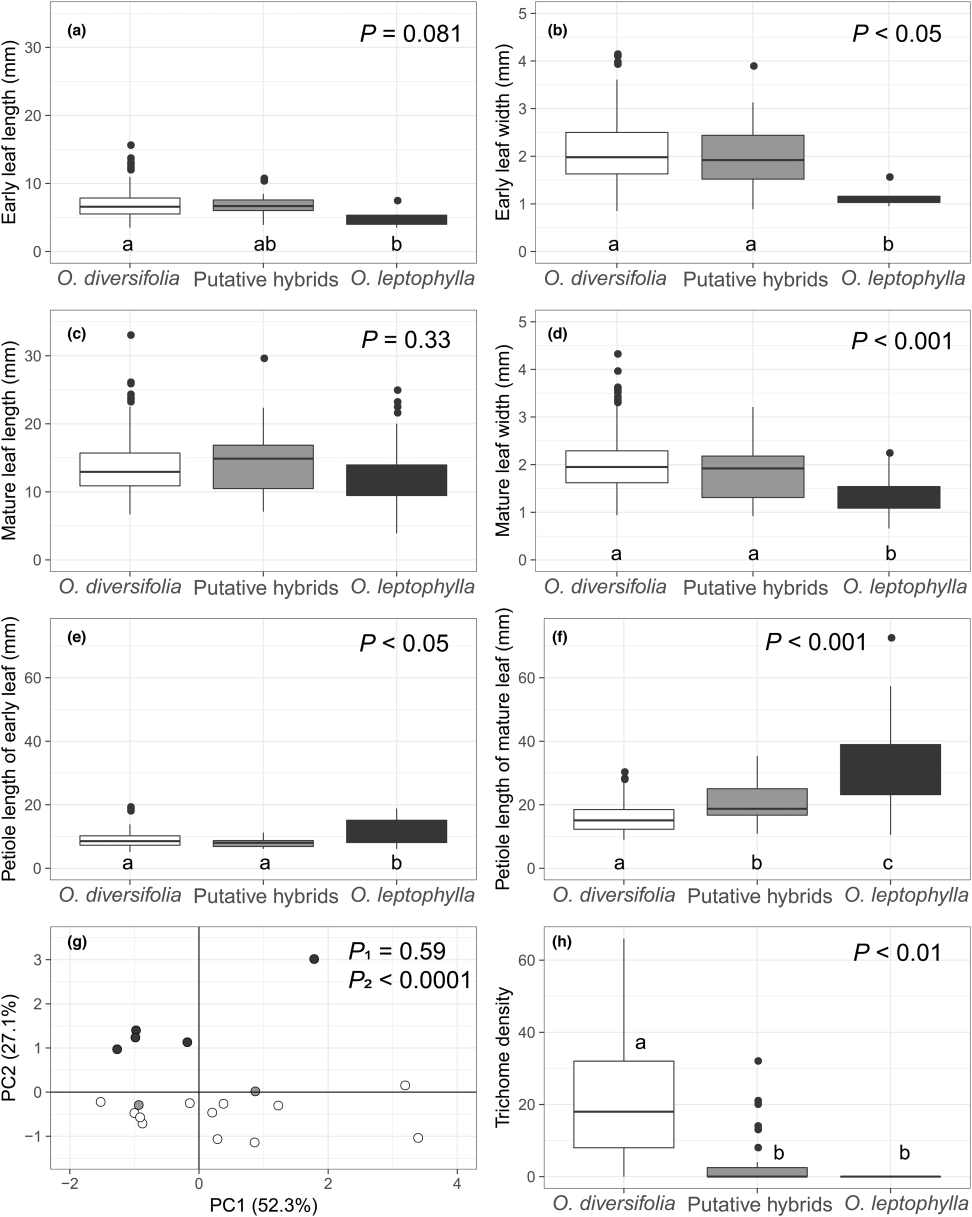
(a–f) Measurements of six leaf‐morphological differences between *Oxytropis diversifolia*, *O. leptophylla*, and putative hybrids. (g) PCA plot showing differentiation at the population level based on six leaf‐morphological traits measured above. White circles, 12 *O. diversifolia* populations; black circles, five *O. leptophylla* populations; gray circles, two populations in the hybrid zone. (h) Trichome density on leaf upper surface, i.e., the number of epidermal hairs counted on a 1 mm × 1 mm surface. *p*‐values indicate the significance of differences among categories in linear mixed‐effects models. Different letters indicate significant differences of pairwise comparisons (*p* < .05, Tukey's HSD test).

Among the six leaf‐morphological traits (Figure 2a–f; Table S5), we found that four traits—including the early leaf width, mature leaf width, petiole length of early leaf, and petiole length of mature leaf—differed significantly between *O. diversifolia*, *O. leptophylla*, and putative hybrids (*p* < .05 in all cases; Figure 2b,d–f). All those four traits were clearly divergent between *O. diversifolia* and *O. leptophylla*. The putative hybrids presented values intermediate between the two parental species for petiole length of mature leaf (Figure 2f); while for other three traits, values of putative hybrids were close to *O. diversifolia* (Figure 2b,d,e). Early leaf length was marginally differed among categories (*p* = .081; Figure 2a), and mature leaf length showed no difference (*p* = .33; Figure 2c). Regarding the PCA performed on the six traits measured above, co‐ordinates on PC1 were highly and positively correlated with four traits measured (Pearson's correlation tests, *r* = 0.75–0.87, *p* < .0001; total contribution 84.1%), while the remaining two variables, mature leaf width and petiole length of mature leaf, were highly correlated with co‐ordinates on PC2 (*r* = −0.69, *p* < .0001 and *r* = 0.82, *p* < .0001, respectively; 70.0%). Populations of the two parental species and putative hybrids were shown to differentiate in the PCA plot (Figure 2g), but it was largely due to the significant effect of PC2 (*p* < .0001; co‐ordinates of putative hybrids were intermediate) rather than PC1 (*p* = .59) (Table S5). Additionally, the trichome density on leaf upper surface was also significantly differed among categories (*p* < .01; Figure 2h, Table S5).

### 3.3 Nuclear microsatellite analyses

#### 3.3.1 Population genetics

Among the 19 locations sampled, 33 out of 1045 (3.2%) within‐population genotypic disequilibria were detected prior to correction for multiple testing, but were not specific for any pairwise loci or population. After Benjamini–Hochberg correction, only one pair of linkage disequilibrium in population PSEV remained significant (Table S6). Significant heterozygote deficits were observed at 1 to 10 loci in each population, and in total 138 out of 209 locus‐specific tests was significant (Table S6). The systematic pattern of heterozygote deficiency found in *O. diversifolia* and *O. leptophylla* is likely due to the self‐compatibility and/or inbreeding (Wang et al., 2021), while in the hybrid zone, the pattern is not significantly different from that of parental species (*p* = .38; Table 2A).

**TABLE 2:** Descriptive statistics of genetic variability based on 11 nuclear microsatellite loci and five cpDNA intergenic regions.

	*O. diversifolia* (12 populations)	Hybrid zone (2 populations)	*O. leptophylla* (5 populations)	*p*‐value
**(A) Nuclear microsatellites**				
*N*_a_	10.8 (2.26) a	11.4 (2.05) ab	7.3 (0.606) b	**<.05**
*H*_o_	0.578 (0.0331) a	0.523 (0.0141) ab	0.495 (0.0379) b	**<.01**
*H*_e_	0.821 (0.0212) a	0.780 (0.0304) a	0.708 (0.0256) b	**<.0001**
*F*_IS_	0.296 (0.0318)	0.327 (0.00707)	0.304 (0.0403)	.38
**(B) cpDNA**				
*S*	9.1 (3.40) a	35.5 (3.54) b	5 (8.94) a	**<.05**
*h*	7.8 (2.12) a	10 (2.83) a	2.4 (0.894) b	**<.01**
*h*_p_	3.1 (1.56) a	1.0 (1.41) ab	0.4 (0.894) b	**<.05**
*Hd*	0.880 (0.0736) a	0.783 (0.0297) a	0.523 (0.132) b	**<.001**
*π*	0.00061 (0.00020) a	0.00394 (0.00010) b	0.00054 (0.00092) a	**<.001**

*Note*: **Nuclear microsatellites:** *N*_a_, number of alleles; *H*_o_, observed heterozygosity; *H*_e_, unbiased expected heterozygosity; *F*_IS_, fixation index. For those genetic diversity parameters, data are presented as mean (*SD*) averaged across populations. **cp DNA:** *S*, number of segregating sites; *h*, number of haplotypes; *h*_p_, number of private haplotypes (i.e., haplotypes that are fixed in only one population); *Hd*, haplotype diversity; *π*, nucleotide diversity. *p*‐values indicate the significance of differences among categories in linear models (for details of the methods, see Wang et al., 2021). Different letters indicate significant differences of pairwise comparisons (*p* < .05, Tukey's HSD test).

At the population level, the genetic diversity parameters (*N*_a_, *H*_o_, *H*_e_) across loci differed significantly between *O. diversifolia*, *O. leptophylla*, and the hybrid zone (Table 2A; Table S7). *O. diversifolia* populations showed significantly higher level of genetic diversity than those of *O. leptophylla*, while the difference between the hybrid zone and two parental species were not obvious (*N*_a_, *H*_o_), or the hybrid zone showed similar estimates as populations of *O. diversifolia* (*H*_e_).

#### 3.3.2 Parent and hybrid assignment

**FIGURE 3:**
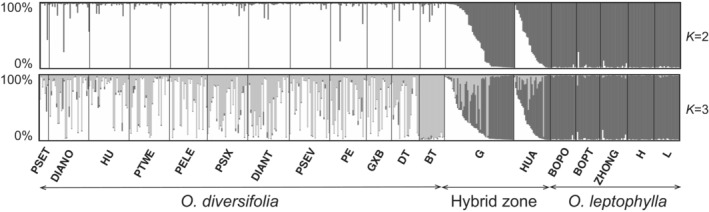
STRUCTURE results for 19 *Oxytropis* populations based on microsatellite dataset (*K* = 2–3). The small black lines separate populations, named at the bottom of the graph. Each individual is a small bar with color coded according to probability of clustering in a particular group. Populations are ordered west to east for each species.

According to the STRUCTURE analysis, the optimal *K* value was 2 (Figure S2), with the two parental species formed distinct genetic groups and the hybrid zone showed genetic admixture (Figure 3). The result of *K* = 3 was also used here to test whether the hybrid individuals formed a separate genetic cluster distinct from either parent, as would be expected if a new hybrid species arisen. At *K* = 3, hybrids did not form a distinct cluster but again showed admixture between the parents; *O. diversifolia* was further divided into two groups (Figure 3).

**FIGURE 4:**
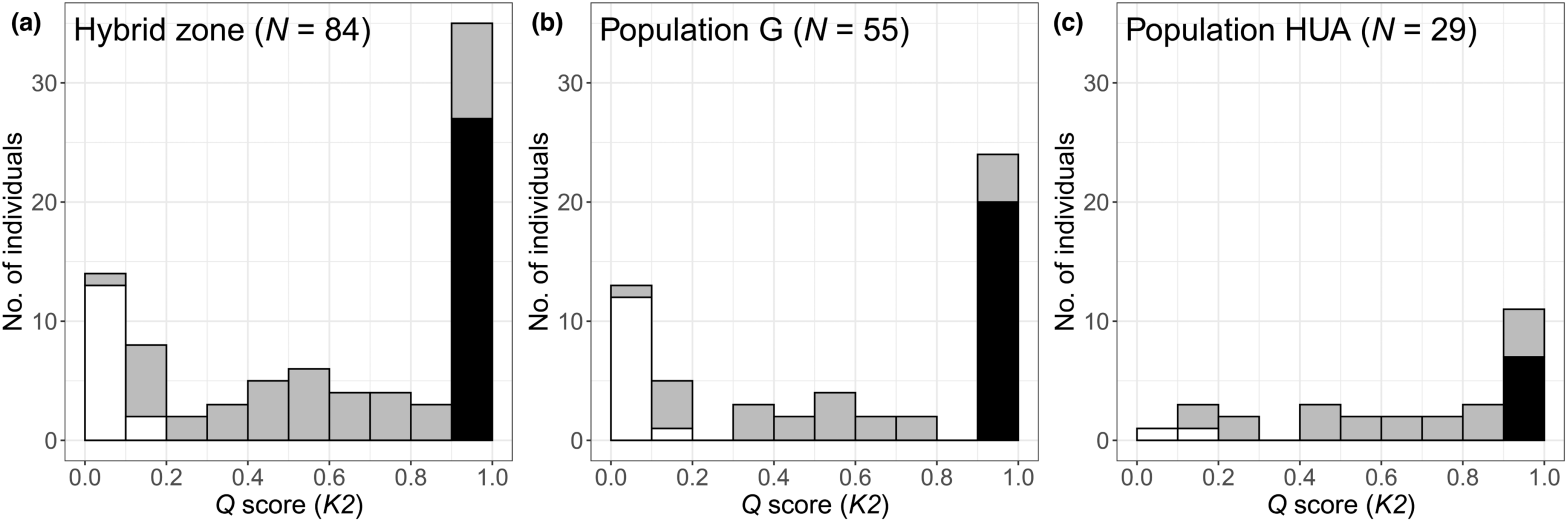
Histogram of microsatellite‐genotype distribution in the hybrid zone, showing a bimodal pattern. (a) Population G and HUA combined; (b) population G only; (C) population HUA only. Individual genotypes identified as parental are shown in white (*Oxytropis diversifolia*) and black (*O. leptophylla*), and hybrids are shaded in gray.

Across the two localities in the hybrid zone, the 84 individuals examined comprised 15 *O. diversifolia* (17.9%), 42 hybrids (50.0%) and 27 *O. leptophylla* (32.1%) (Table 3, Table S4). No significant difference was detected between the number of hybrid individuals with >50% *O. diversifolia* nuclear germplasm and those with >50% *O. leptophylla* (Table 3). However, the histogram of *Q* scores showed a lower frequency of intermediate genotypes and a higher frequency of pure parental genotypes, yielding a bimodal pattern (Hartigans' dip test, *D* = 0.074, *p* < .001; Figure 4a). When analyzing two populations separately, the bimodal pattern is still significant in population G (*D* = 0.109, *p* < .0001; Figure 4b), but not in population HUA (*D* = 0.049, *p* = .88; Figure 4c). Furthermore, there were more “pure” genotypes of *O. leptophylla* than those of *O. diversifolia* in the hybrid zone (*χ* ^2^ = 9, df = 1, *p* < .01), and this remained true when G and HUA analyzed separately (*χ* ^2^ = 3.3, df = 1, *p* = .07 and *χ* ^2^ = 8.3, df = 1, *p* < .01, respectively).

**TABLE 3:** Number of parental and hybrid individuals assigned based on nuclear microsatellite data

Population	No. 1^a^	Assignment based on nuclear microsatellites^b^				No. 2 ^c^	cpDNA haplotype	
		OD	Hybrids	OL	Hybrids (*Tq* < 0.50): (*Tq* > 0.50)		All	Hybrids
							(OD:OL)	(OD:OL)
**(1) *O. diversifolia***	**321**	**299**	**22**	**0**	**19:3**	**154**	**154:0**	**12:0**
PSET	7	6	1	0	0:1	6	6:0	−
DIANO	32	27	5	0	4:1	15	15:0	3:0
HU	32	29	3	0	3:0	15	15:0	1:0
PTWE	32	32	0	0	−	15	15:0	−
PELE	30	30	0	0	−	15	15:0	−
PSIX	32	30	2	0	2:0	17	17:0	2:0
DIANT	33	31	2	0	2:0	15	15:0	1:0
PSEV	32	31	1	0	1:0	15	15:0	−
PE	29	27	2	0	1:1	15	15:0	1:0
GXB	20	20	0	0	−	8	8:0	−
DT	22	19	3	0	3:0	10	10:0	3:0
BT	20	17	3	0	3:0	8	8:0	1:0
**(2) Hybrid zone**	**84**	**15**	**42**	**27**	**17:25 ns**	**64**	**26:38 ns**	**17:12 ns**
G	55	13	22	20	10:12 ns	43	16:27 ns	7:8 ns
HUA	29	2	20	7	7:13 ns	21	10:11 ns	10:4 ns
**(3) *O. leptophylla***	**102**	**0**	**3**	**99**	**0:3**	**43**	**0:43**	**0:2**
BOPO	20	0	0	20	−	8	0:8	−
BOPT	19	0	1	18	0:1	8	0:8	0:1
ZHONG	22	0	2	21	0:2	9	0:9	0:1
H	21	0	0	21	−	9	0:9	−
L	20	0	0	20	−	9	0:9	−

*Note*: For the hybrid zone, *χ* ^2^ tests were performed between the number of hybrids having >50% *O. diversifolia*‐like germplasm (*Tq* < 0.50) or >50% *O. leptophylla*‐like germplasm (*Tq* > 0.50), and between the ratio of *O. diversifolia* (OD) to *O. leptophylla* (OL) cpDNA haplotypes for all sequenced individuals and putative hybrids.

Abbreviation: ns, not significant.

^a^ Number of individuals genotyped at 11 nuclear microsatellite loci.

^b^ Final assignment was determined based on the criteria that two out of the three methods (STRUCTURE, hybrid index, NEWHYBRIDS) yielded consistent results.

^c^ Number of randomly selected individuals sequenced for five cpDNA fragments.

Concerning the morphology of individuals in the hybrid zone, most individuals genetically assigned as *O. diversifolia* or *O. leptophylla* exhibited the relevant morph (37 out of 42, 88.1%; Table S4). However, the morphology of hybrids was quite diverse: hybrid phenotypes ranged from directly intermediate (e.g., Figure 1e,f; 19 out of 31 individuals, 61.3%) to either parental species phenotypes (12 out of 31 individuals, 38.7%) (considering both flowers and leaves; Table S4). It is worth noting that, hybrids were more frequent to have leaf‐shape phenotype of *O. diversifolia* than that of *O. leptophylla* (19 vs.7, *χ* ^2^ = 5.54, df = 1, *p* = .019; the remaining five individuals with intermediate 3–5 leaflets), but there was no difference for the floral phenotype of hybrids to be either parental species (14 vs.11, *χ* ^2^ = 0.36, df = 1, *p* = .55; the remaining six individuals with mosaic flower color and/or intermediate peduncle length).

The program NEWHYBRIDS can further assign hybrids to four different hybrid categories: *F*_1_s, *F*_2_s, backcrosses to *O. diversifolia*, and backcrosses to *O. leptophylla*. No individual detected had greater than a 9.5% chance of being classified as *F*_1_s (Table S4). This analysis struggled to assign individuals to specific classes with any confidence, which is probably due to the lack of species‐diagnostic loci (Anderson & Thompson, 2002), and could also be an expected outcome when hybrid derivatives of the third generation or beyond, and other complex intermediate hybrid derivatives are present (Yan et al., 2017).

### 3.4 Chloroplast DNA sequence analyses

The concatenated cpDNA dataset had a total alignment length of 3918 bp. Across all 261 sequences, 57 haplotypes were identified, characterized by 80 variable sites, of which 45 were parsimony informative. The 197 individuals from putatively “pure” populations of two parental species were fixed for distinct haplotypes (Table 3), with H1–H51 in *O. diversifolia* and H54–H57 in *O. leptophylla*, respectively (Figure S3). Two haplotypes, H52 and H53, were only found in the hybrid zone, but they were derivatives of *O. diversifolia* haplotypes H42 and H6, respectively (Figure S3). Consequently, they can be *O. diversifolia* haplotypes miss detected in putatively “pure” populations. Significant cytonuclear disequilibrium was detected in both population G and HUA (*p* < .01, Table S6). The genetic diversity estimates of *S* and *π* were highest in the hybrid zone (Table 2B; Table S8). For other estimates of *h*, *h*_p_, and *Hd*, the difference between the hybrid zone and two parental species were not obvious (*h*_p_) or the hybrid zone showed similar estimates as populations of *O. diversifolia* (*h*, *Hd*) (Table 2B; Table S8).

Across the two localities in the hybrid zone, the 64 individuals sequenced had either *O. diversifolia* haplotypes (26 individuals, 40.6%) or *O. leptophylla* haplotypes (38 individuals, 59.4%) (Table 3; Figure S3). Of these, 29 individuals were classified as hybrids with nuclear analyses (Table S4): 17 possessed *O. diversifolia* haplotypes whereas 12 possessed *O. leptophylla* haplotypes, resulting in a 17:12 ratio, not differing significantly from the 1:1 expectation for no gender bias (*χ* ^2^ = 0.86, df = 1, *p* = .35; Table 3).

### 3.5 Micro‐ and macrohabitat differentiation

#### 3.5.1 Microhabitat differentiation

**FIGURE 5:**
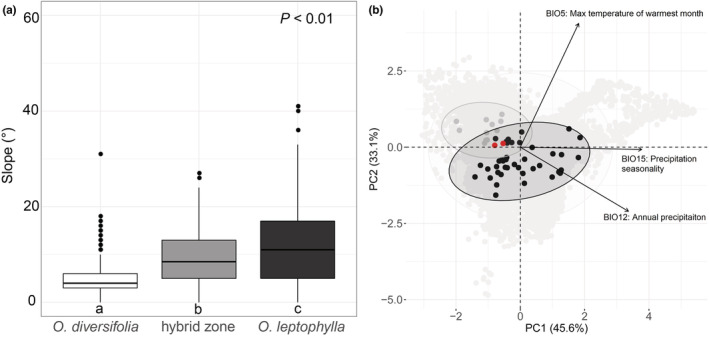
Micro‐ and macrohabitat differentiation. (a) Boxplot showing the individual‐level difference of a microhabitat variable “slope”. *p*‐values indicate the significance of difference among categories in linear mixed‐effects models. Different letters indicate significant differences of pairwise comparisons (*p* < .05, Tukey's HSD test). (b) PCA plot showing macroclimatic differentiation for localities of *Oxytropis diversifolia*, *O. leptophylla*, and the hybrid zone based on three selected contemporary bioclimatic variables. Points represent principal component scores on the first two principal component axes. Light gray circles, background (10,000 localities); gray circles, *O. diversifolia* (12 localities); black circles, *O. leptophylla* (40 localities); red circles, the hybrid zone (2 localities). Ellipsoid is defined by a 95% confidence interval.

We found that values of slope (°) in the hybrid zone (10.1 ± 6.41, mean ± *SD*) were intermediate between the two parental species (4.95 ± 3.63 in *O. diversifolia* and 12.9 ± 9.74 in *O. leptophylla*, respectively) (*p* < .01; Figure 5a, Table S9). However, the percent of total vegetation cover, the percent of rocky ground, and the percent of bare ground did not differ among categories (*p* = .15–.69; Table S9).

#### 3.5.2 Macrohabitat differentiation

Seven out of the 19 bioclimatic variables showed significant differentiation, with localities of the hybrid zone possessed intermediate values between those of the two parental species (Table S10). We retained max temperature of warmest month (BIO05), annual precipitation (BIO12), and precipitation seasonality (BIO15) for PCA. The majority localities of *O. leptophylla* had distinct macroclimatic niche from *O. diversifolia*, and the two localities of the hybrid zone fell in the overlapping area (Figure 5b).

However, concerning soil characteristics, soil pH was marginally differentiated among categories (8.31 ± 0.17 in *O. diversifolia*, 8.37 ± 0.10 in the hybrid zone, and 7.83 ± 0.73 in *O. leptophylla*, respectively) (one‐way ANOVA, *p* = .055; Figure S4). The majority of localities (43 out of 54, 80.0%) were alkaline soil with pH between 8.0 and 8.5. The change of soil type was gradual. For *O. diversifolia*, five localities in the west (PSET, DIANO, HU, PTWE, PSIX) possessed Calcisols, and five localities to the east (DIANT, PSEV, PE, GXB, DT) were Kastanozems; the two localities in the hybrid zone were also Kastanozems; For *O. leptophylla*, the two soil types most common were Kastanozems (17 out of 40 localities, 42.5%) and Chernozems (6 out of 40 localities, 15%) (Figure S5a). Kastanozems in *O. diversifolia* and hybrid zone were almost calcic Kastanozems (KSk), while those in *O. leptophylla* were almost haplic Kastanozems (KSh) and luvic Kastanozems (KSl) (Figure S5b). Similarly, the associated plant species in 19 localities that we surveyed were coupled to the gradual change of both macroclimatic and soil characteristics (Table S11).

### 3.6 Geographical cline analysis

**FIGURE 6:**
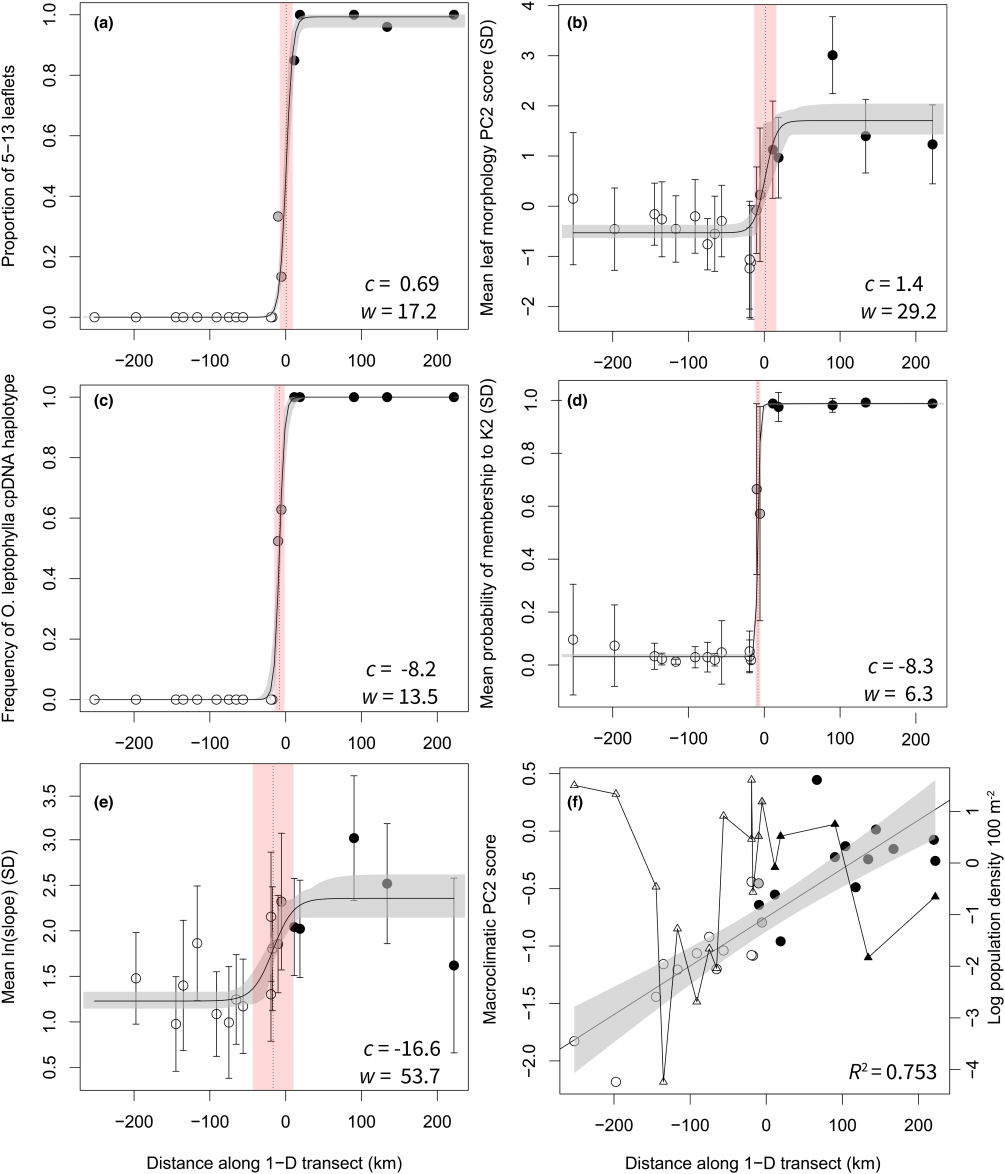
Variation in leaf morphological characteristics, neutral genetic variation, habitat, and population density across the collapsed transect. (a) Leaf‐shape phenotype; (b) leaf morphology (PC2 scores); (c) cpDNA haplotype; (d) nuclear microsatellite genotype (*Q*_*K2*_ scores generated from STRUCTURE); (e) microhabitat (log transformed values of slope); (f) macrohabitat (macroclimatic PC2 scores, circles) and population density (log individuals per 100 m^2^, tringles). The maximum‐likelihood cline (with 95% credible cline region) in a‐e are fitted using the MCMC algorithm. The vertical dashed line represents the geographic cline center (*c*). The shaded red area represents the estimated cline width (*w*). White symbols, *O. diversifolia*; black symbols, *O. leptophylla*; gray symbols, localities in the hybrid zone.

We plotted the maximum‐likelihood cline for the best‐fit model for each morphological, genetic, and environmental variable (Figure 6a–f). All variables, except for macroclimatic PC2 score, exhibited sigmoidal variation along the 1D transect. Estimated cline centers and the corresponding cline widths were shown in Figure 6a–e. See Table S12 for details of the cline parameters. Instead of a sigmoidal cline, the macroclimatic PC2 score exhibited linear variation along the transect (Multiple *R* ^2^ = 0.753, *p* < .0001), and localities of the hybrid zone possessed intermediate values (Figure 6f). Population density showed considerable variation, and the number of individuals ranged from 0.014 to 5.0 per 100 m^2^ among 19 sites (Figure 6f; Table S1). However, there was no significant difference inside and outside the hybrid zone (*O. diversifolia* = 1.61 ± 1.87, mean ± *SD*; the hybrid zone = 2.48 ± 1.13; *O. leptophylla* = 1.08 ± 0.810; one‐way ANOVA, *F*_2,16_ = 0.69, *p* = .52).

We then tested for cline coincidence and concordance based on cpDNA haplotypes, nuclear microsatellite genotypes and microhabitat data. A model in which three clines were constrained to have the same width did not fit the data as well as a model in which the cline centers and widths were allowed to vary independently (InLikelihood = −115.8, *k* = 27, AIC = 285.6 vs. InLikelihood = 525.5, *k* = 29, AIC = −993.0). However, a model in which clines of cpDNA haplotypes and microhabitat constrained to the same center exhibited a better fit (InLikelihood = 613.3, *k* = 28, AIC = −1170.6). These results indicated that genetic clines and the environmental cline were coincident, but genetic clines were narrower than the environmental cline. Moreover, we found that the leaf‐shape cline showed shifted cline center when compared with genetic clines (clines of leaf shape and cpDNA haplotypes: center constrained AIC = 346.2 vs. unconstrained AIC = 309.0; clines of leaf shape and microsatellite genotypes: center constrained AIC = −2005.6 vs. unconstrained AIC = −2039.6).

## 4 DISCUSSION

Hybrid zones generated through secondary contact between closely related species have been widely highlighted for the interest in understanding the evolutionary processes (Barton & Hewitt, 1985; Harrison, 1993). However, the number of plant hybrid zones detected and studied genetically was rather limited (reviewed in Abbott, 2017). In the present study, we integrated morphological, molecular, and ecological analyses to investigate the hybrid zone generated between parapatric distributed *O. diversifolia* and *O. leptophylla* in Nei Mongol, China. We found that most leaf‐morphological traits measured significantly differ between two parental species, and putative hybrids show either intermediacy or a bias to *O. diversifolia*. The two parental species are clearly separable based on data of nuclear microsatellites and cpDNA haplotypes; populations in the hybrid zone exhibits a bimodal pattern, consisting of both parental forms and a range of hybrid admixture types with variable admixture proportions. Finally, the hybrid zone is coupled to ecological transitions of both microhabitat (i.e., the slope) and macroclimatic conditions. However, the genetic clines are significantly narrower than the environmental cline. These results indicate that endogenous selection can be primarily responsible for maintaining the hybrid zone, while local adaptation accounts for the position of the zone. Below we discuss the structure of hybrid zone in detail and tentatively propose potential effects of hybridization.

### 4.1 Endogenous selection primarily responsible for maintaining the narrow hybrid zone

Hybrid zones are often formed after the secondary contact of two divergent parental forms, and some previous definitions restricted the term hybrid zone to narrow clines (Barton & Hewitt, 1985), although mosaic hybrid zones can also be common (Abbott, 2017). In this study, the two *Oxytropis* species are separated by a narrow hybrid zone that can be maintained by a balance between dispersal and divergent selection. This inference is based on the shape of the phenotypic and genetic transition (i.e., sigmoidal clines), the narrow width relative to the dispersal ability (Wang et al., 2021), and the elevated variance at the center (Barton & Hewitt, 1985; Stankowski et al., 2021). However, solely from the sigmoidal cline, it is difficult to distinguish between selection against hybrids (i.e., “endogenous” selection) and adaptation to different environments (i.e., “exogenous” selection), because endogenous and exogenous selection can generate clines of similar shape (Barton & Gale, 1993; Barton & Hewitt, 1989; Kruuk et al., 1999).

Taken our results together, we suggest that endogenous selection can be more important. First, although the genetic clines coincide with the environmental cline, the former is significantly narrower than the latter. This is particularly evident for the comparison between genetic clines and the macroclimatic gradient. The observed pattern is consistent with the prediction of tension zone model, in which endogenous selection can create a sufficiently strong barrier to gene flow independent of habitat (Barton & Hewitt, 1985, 1989). Second, we investigated clinal variation across the hybrid zone in a variety of characters, either morphological or genetical, which show close concordance as parallel gradients. If each allele was to respond directly to the environment, such geographic concordance would not be expected (Barton & Hewitt, 1985). Finally, the evidence for endogenous selection against hybrids is usually in the form of hybrid inviability, sterility or abnormality in the field or laboratory (Stankowski et al., 2021). Due to the perennial feature and field work limitation, we are not able to provide a direct measure of hybrid inviability or sterility (but see Milne & Abbott, 2008 and Yan et al., 2019 for example). However, we did observe abnormality of leaf characteristics in the hybrid zone (Table S4).

Because tension zones can be maintained independent of environmental conditions, they can move from place to place. There are two main explanations for the position of hybrid zone. The first hypothesis is the “low‐density troughs”. Environmental features like valleys, rivers, ridges and wood cause density to vary over orders of magnitude, and moving hybrid zones have the interesting property of tending to rest in regions of low density (Hewitt, 1988). The alternative hypothesis suggests that tension zones have a tendency to become trapped by, and therefore to coincide with, exogenous barriers due to ecological selection (Bierne et al., 2011). In our study, none of the clines are positioned in an area of low density. Instead, genetic clines are coupled to ecological transitions. Theoretical simulation indicates that coupling is more likely with moderate exogenous selection, as it results in very wide cline (e.g., latitudinal clines) able to trap the endogenous tension zone from a remote distance (Bierne et al., 2011). In our case, the microhabitat cline is wider than genetic clines, and the macroclimatic transition is even wider along the longitude. Both of them can produce moderate exogenous selection to trap the tension zone.

### 4.2 Potential effects of hybridization

A pronounced feature of the hybrid zone detected in our study is the bimodal pattern of genotypic distribution. Historical literature review suggests that both bimodal and unimodal hybrid zones have been shown to exhibit postzygotic isolating mechanisms, which may be either intrinsic or extrinsic. However, only bimodal zones are associated strongly with prezygotic barriers (Jiggins & Mallet, 2000). The flowering phenology of *O. diversifolia* is slightly earlier than *O. leptophylla* (April to May vs. May to June). Although the floral structure of both species is quite the same and they can be bee‐pollinated, the difference in flower color is obvious (pale yellow vs. purplish red). Because both species are not rewardless (they produce nectar, H. Wang, personal observation), the distinct flower color can somehow prevent interspecies pollen transfer.

Moreover, the bimodal distribution seems to be asymmetrical. Asymmetric hybrid zones occur when strong asymmetries in gene flow and barrier strength promote hybridization and introgression in one direction, such that gene flow occurs only from one species to another (Abdelaziz et al., 2021; Pickup et al., 2019). In our case, we suggest that biased introgression from *O. leptophylla* to *O. diversifolia* is plausible. Actually, we did find that in the hybrid zone, hybrids are more frequent to have leaf shape and morphology of *O. diversifolia* than that of *O. leptophylla*. However, we are cautious that the microsatellite loci we used are rather limited, and they are not species‐specific. It is hard to unambiguously assign genetic variants to parental taxa, and thus fully characterize the hybrid origin and hybridization history of plants in the hybrid zone. For example, there might be shared variation among parental taxa. Further characterization of these hybrids using species‐specific nuclear markers should be warranted (e.g., SNPs).

Our data also indicated potential cytoplasmic introgression. We found that two rare cpDNA haplotypes of *O. leptophylla*, i.e., H56 and H57 detected in the allopatric population ZHONG, are distinct from major haplotypes of this species but close to *O. diversifolia* haplotypes (Figure S3), while the two individuals possessing those haplotypes are still of *O. leptophylla* morphology and nuclear germplasm. This may be due to an ancient “chloroplast capture” event through hybridization and introgression, which frequently happens in plants (Rieseberg, 1995). Chloroplast capture is probably one of the most common causes of phylogenetic incongruence between nuclear and plastid gene tree (Wendel & Doyle, 1998). It has been reported in phylogenetic studies involving deep relationships among different genera (e.g., Liu et al., 2017), and also detected at lower taxonomic ranks in population and/or species‐level studies (e.g., Wendel et al., 1991). Moreover, in the hybrid zone we found six individuals with the nuclear germplasm of *O. diversifolia* but cpDNA haplotypes of *O. leptophylla* and vice versa (Table S4), suggesting that there might be ongoing cytoplasmic introgression.

Introgression is emerging as an important source of novel genetic variation, alongside with standing variation and mutation, and it can be adaptive when such introgressed alleles are maintained by natural selection (termed “adaptive introgression”, Abbott et al., 2013; Suarez‐Gonzalez et al., 2018). A previous study revealed that the clinal variation in leaf shape in *O. diversifolia* can be attribute to natural selection imposed by spatially varying macroclimatic factors (Wang et al., 2021). It would be interesting to test whether leaf‐shape polymorphism in *O. diversifolia* was obtained through hybridization and introgression from *O. leptophylla* to the 1‐leaflet phenotype. The detection of nuclear introgression requires makers that are strongly species‐specific, and again, microsatellites used in our study is not satisfying. Several studies have applied next‐generation sequencing and revealed that introgression transfers adaptive alleles. For example, *Arabidopsis arenosa* became adapted to drought and toxic levels of soil minerals in serpentine soil through introgression of alleles from *A. lyrata* (Arnold et al., 2016). Similarly, introgression from *Cupressus gigantea* into a related species *C. duclouxiana* likely aided the latter species to extend its range by colonizing cooler and drier mountain habitats during postglacial periods (Ma et al., 2019).

## 5 CONCLUSION

In summary, we have found evidence of bimodal hybrid zone connecting two parapatric *Oxytropis* species; the geographic cline analysis indicated that the hybrid zone can be maintained by a balance between dispersal and selection, with the latter primarily being endogenous. We further conclude that the probable outcome of hybridization is introgression. A potential hypothesis to be tested is adaptive introgression, i.e., whether leaf‐shape polymorphism in *O. diversifolia* was obtained through introgression from *O. leptophylla*. Future work applying restriction site associated DNA sequencing (RAD‐Seq) and/or whole‐transcriptome sequencing (RNA‐Seq) is necessarily important.

## AUTHOR CONTRIBUTIONS

**Hui Wang:** Conceptualization (equal); data curation (lead); formal analysis (lead); funding acquisition (lead); resources (equal); supervision (lead); writing – original draft (lead); writing – review and editing (lead). **Xin‐Nuo Li:** Data curation (supporting); formal analysis (supporting); investigation (equal). **Song‐Hua Mo:** Data curation (supporting); formal analysis (supporting); investigation (equal). **Min Wang:** Investigation (equal). **Pei‐Liang Liu:** Conceptualization (equal); formal analysis (supporting); resources (equal). **Qin Li:** Data curation (supporting); formal analysis (supporting); writing – original draft (supporting); writing – review and editing (supporting). **Zhao‐Yang Chang:** Conceptualization (equal); resources (equal); supervision (supporting).

## CONFLICT OF INTEREST

The authors declare no conflict of interest.

## Supporting information


Supinfo

TableS4 ‐ S11

## Data Availability

Chloroplast sequences: GenBank accessions MW511796 ‐ MW512115. Morphology, microsatellite genotypes, microhabitat, and macroclimatic data are available in Dryad (https://doi.org/10.5061/dryad.2547d7wtd).
