# Supplementary material for: Tension zone trapped by exogenous cline: Analysis of a narrow hybrid zone between two parapatric Oxytropis species (Fabaceae)
Source: Ecol Evol. 2022 Sep 20;12(9):e9351. doi: 10.1002/ece3.9351 (PMC9487875; doi:10.1002/ece3.9351)
Supplement: Supplementary file 1 — Supinfo [file ECE3-12-e9351-s001.docx]

**Supporting Information**

**Figure S1** Example of a microhabitat plot (40 cm × 40 cm). Scale bar = 5 cm.


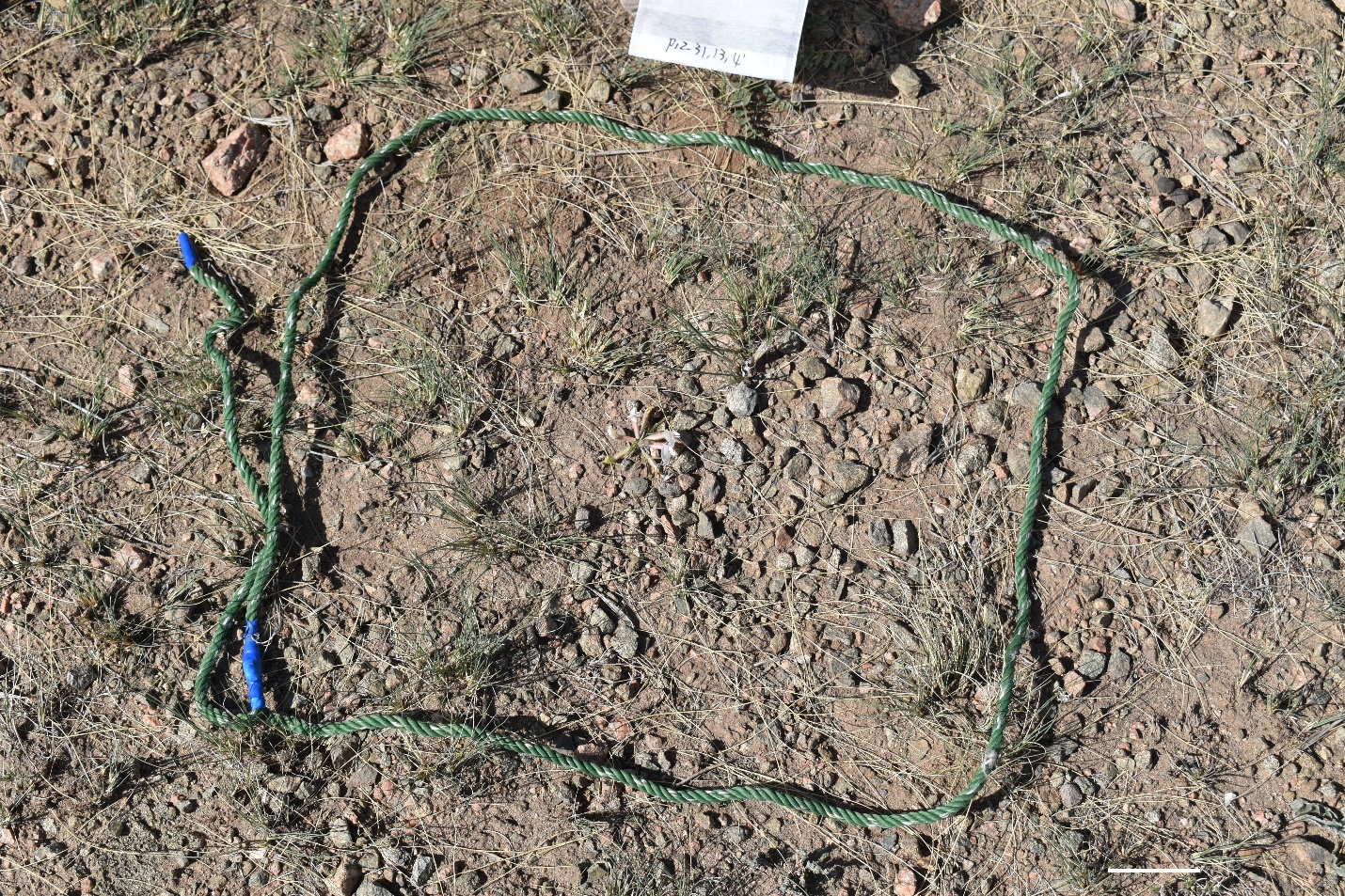


**Figure S2** Bayesian inference of the number of clusters (*K*), from STRUCTURE analysis of nuclear microsatellite dataset for all 19 populations combined. The optimum number of clusters (the *K* that best fit the data) was estimated using the distribution of delta *K* based on L(*K*) (Evanno et al., 2005). The best *K* inferred was 2.


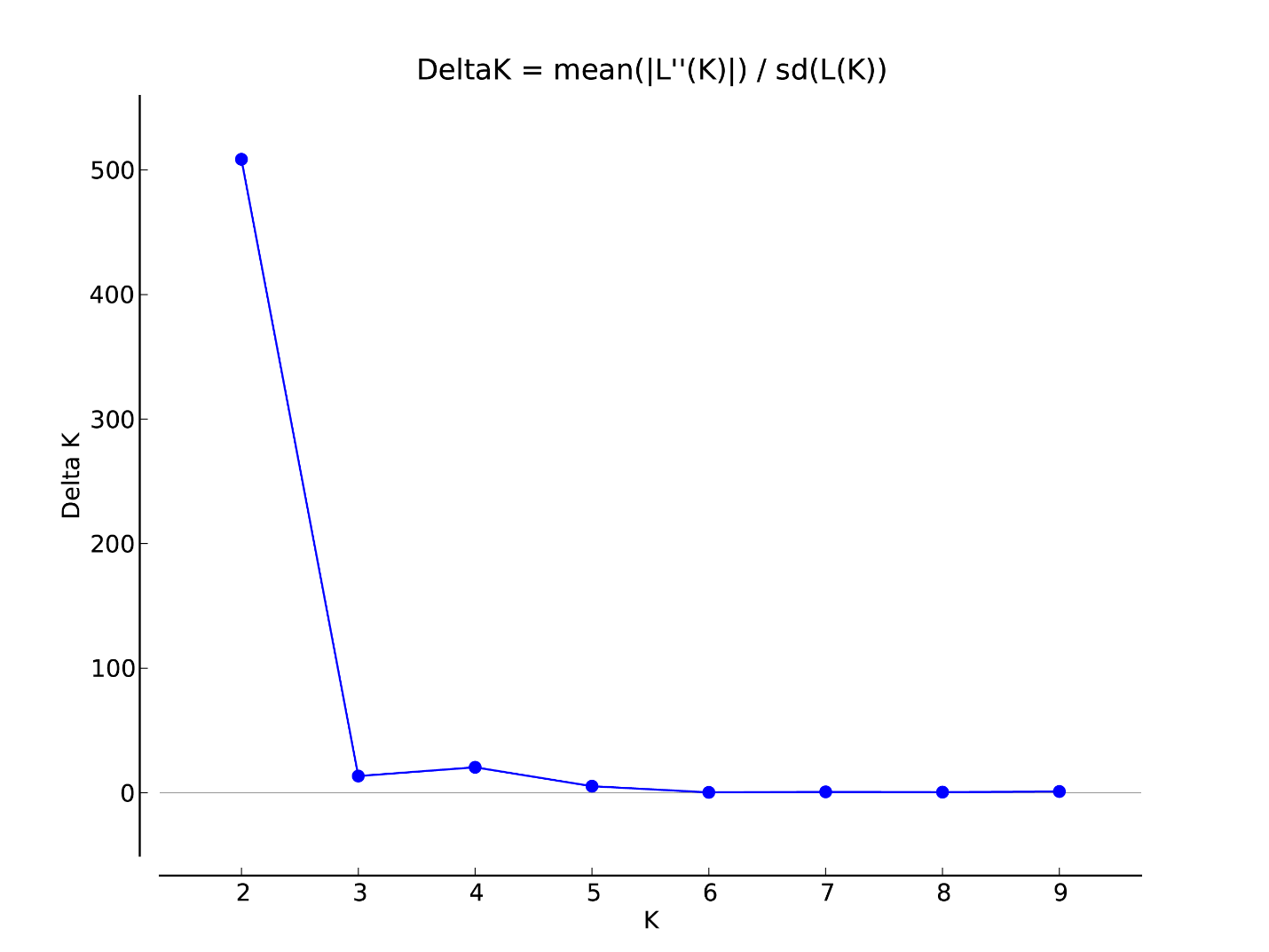


**Figure S3** Network-derived genealogical relationships of the 57 cpDNA haplotypes. These haplotypes were identified from 154 individuals sampled from 12 *Oxytropis diversifolia* populations, 43 individuals from five *O. leptophylla* populations, and 64 individuals from two populations in the hybrid zone.


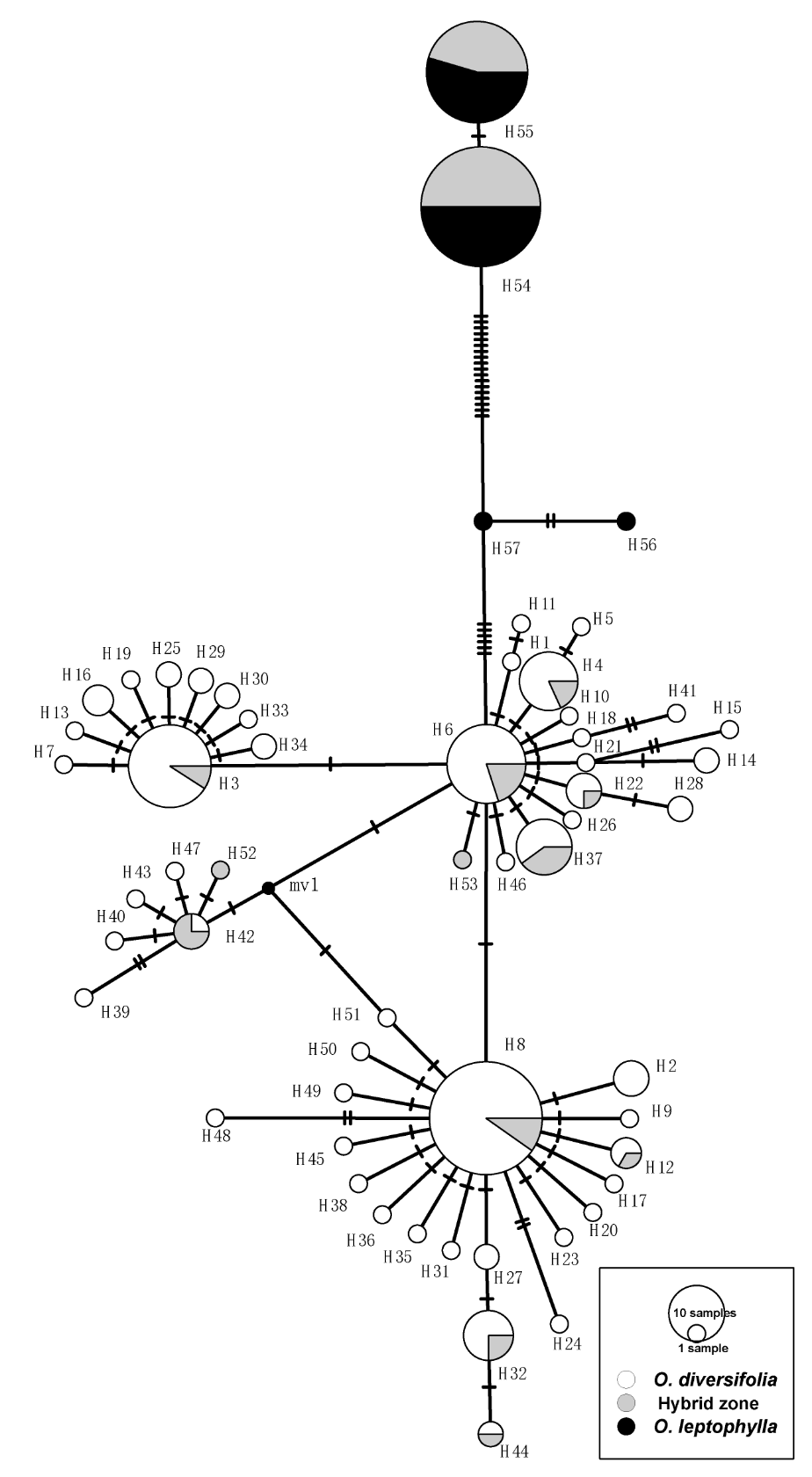


**Figure S4** Soil pH characteristics of the 54 *Oxytropis* localities. (A) Boxplot showing the soil pH differentiation between two parental species and the hybrid zone. *P*-values indicate the significance of difference among categories in one-way ANOVA. (B) Scatterplot of soil pH values along the collapsed transect. White circles, *O. diversifolia* (12 localities); black circles, *O. leptophylla* (40 localities); grey circles, the hybrid zone (2 localities).


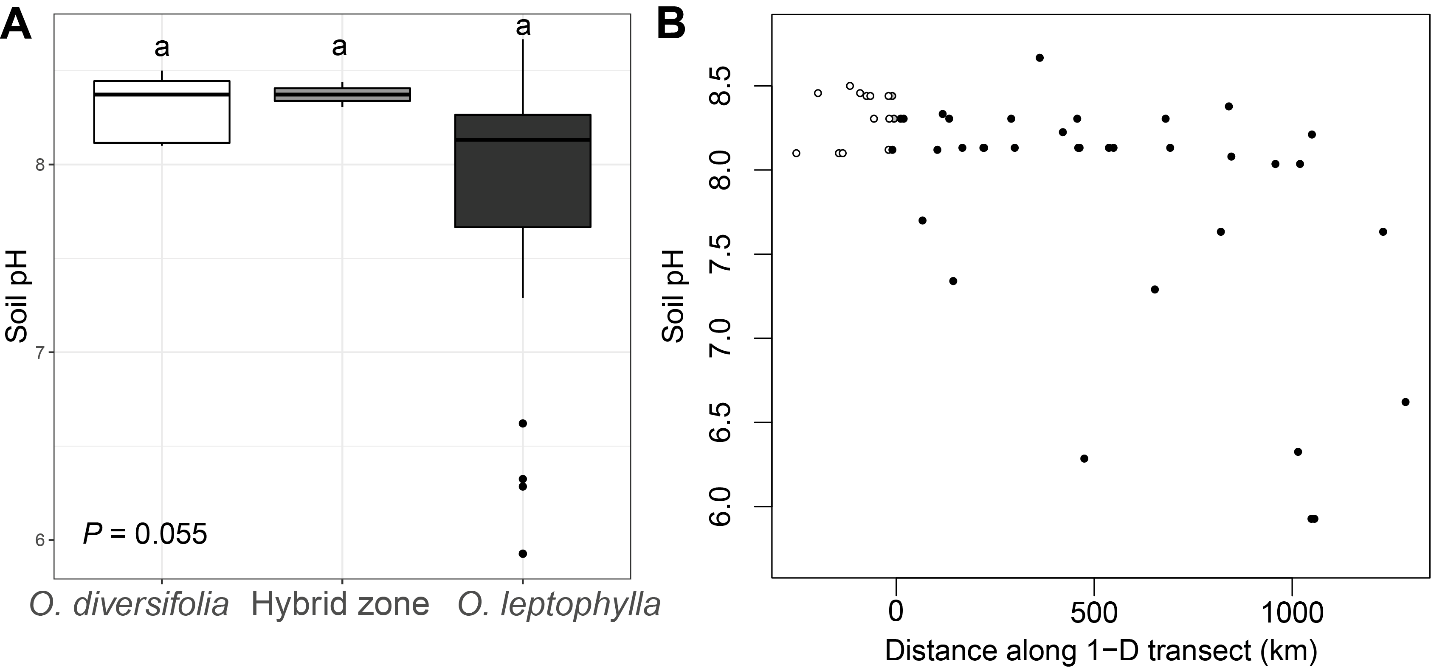


**Figure S5** (A) Reference soil group counts and (B) Kastanozems category counts of the 54 *Oxytropis* localities. KSk, Calcic Kastanozems; KSh, Haplic Kastanozems; KSl, Luvic Kastanozems.


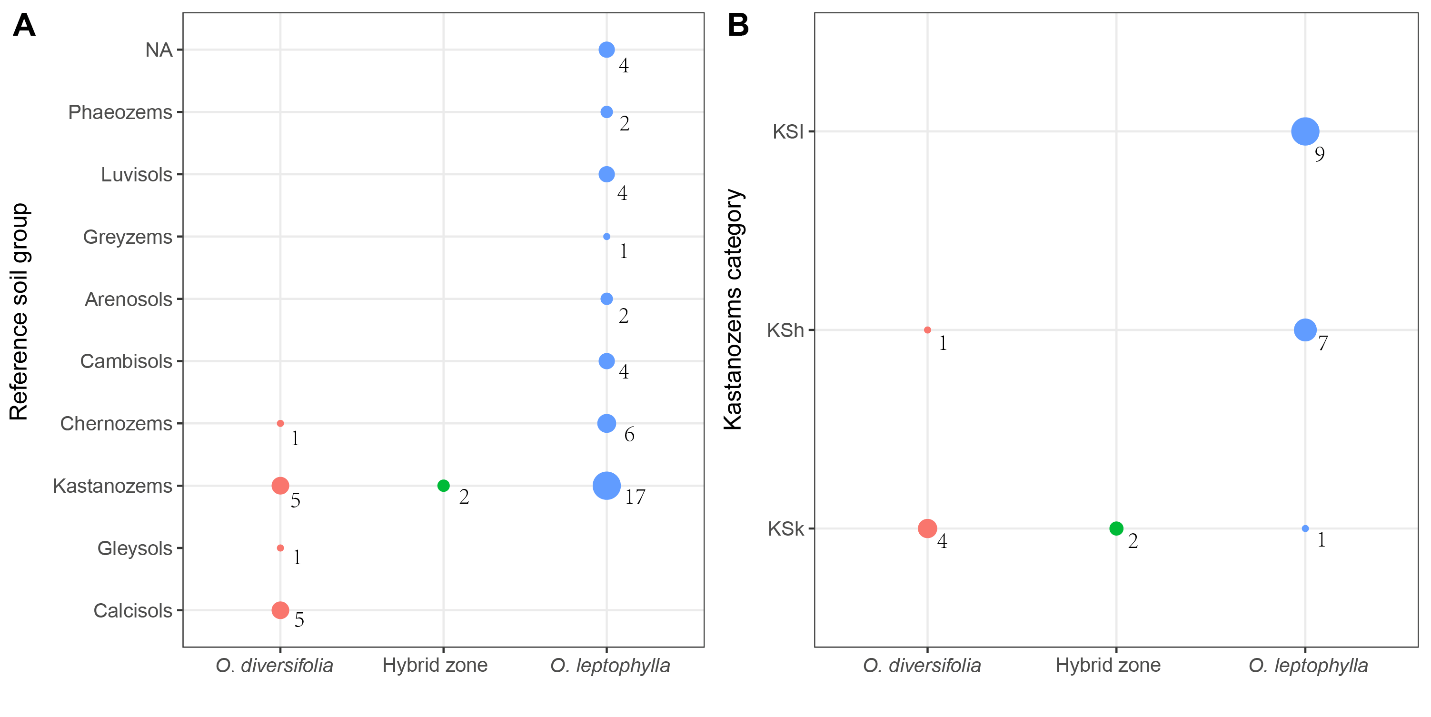


**Table S1** Characteristics of the 19 *Oxytropis* populations sampled. Populations are sorted by increasing longitude (from west to east in Fig. 1G) for each species and hybrid zones, respectively.

| **No.** | **Species** | **Pop.** | **Collection locality** | **Voucher no.^a^** | **Year** | **GPS** | | **Alt.**  **(m)** | **Size^b^** | **Density^c^** | **Phenotype proportion^d^** | | | | |
| --- | --- | --- | --- | --- | --- | --- | --- | --- | --- | --- | --- | --- | --- | --- | --- |
|  |  |  |  |  |  | **Lat.**  **(°N)** | **Long.**  **(°E)** |  |  |  | **1 leaflet** | **1-3 leaflets** | **3 leaflets** | **3-5 leaflets** | **5-13 leaflets** |
| 1 | *O. diversifolia* | PSET | Urad Houqi,  Nei Mongol | Chang2016076 | 2016 | 41.4775 | 106.9509 | 1604 | 9 | 4.5 | 44.4% (4) | 22.2% (2) | 33.4% (3) | 0 | 0 |
| 2 | *O. diversifolia* | DIANO | Urad Zhongqi,  Nei Mongol | Chang2017030 | 2017 | 41.5207 | 107.6290 | 1480 | 76 | 3.8 | 5.3% (4) | 26.3% (20) | 68.4% (52) | 0 | 0 |
| 3 | *O. diversifolia* | HU | Urad Zhongqi,  Nei Mongol | Chang2017034 | 2017 | 41.5694 | 108.3052 | 1319 | 57 | 0.63 | 8.8% (5) | 63.1% (36) | 28.1% (16) | 0 | 0 |
| 4 | *O. diversifolia* | PTWE | Urad Zhongqi,  Nei Mongol | Chang2016029 | 2017 | 41.6102 | 108.4500 | 1441 | 36 | 0.014 | 25.0% (9) | 33.3% (12) | 41.7% (15) | 0 | 0 |
| 5 | *O. diversifolia* | PELE | Urad Zhongqi,  Nei Mongol | Chang2016027 | 2017 | 41.5265 | 108.6436 | 1299 | 45 | 0.28 | 35.5% (16) | 37.8% (17) | 26.7% (12) | 0 | 0 |
| 6 | *O. diversifolia* | PSIX | Urad Zhongqi,  Nei Mongol | Chang2016022 | 2017 | 41.4911 | 108.9556 | 1352 | 170 | 0.068 | 15.9% (27) | 18.2% (31) | 65.9% (112) | 0 | 0 |
| 7 | *O. diversifolia* | DIANT | Urad Zhongqi,  Nei Mongol | Chang2016020 | 2017 | 41.4304 | 109.1337 | 1453 | 121 | 0.19 | 20.7% (25) | 9.1% (11) | 70.2% (85) | 0 | 0 |
| 8 | *O. diversifolia* | PSEV | Urad Zhongqi,  Nei Mongol | Chang2016023 | 2017 | 41.4224 | 109.2560 | 1493 | 98 | 0.13 | 23.5% (23) | 35.7% (35) | 40.8% (40) | 0 | 0 |
| 9 | *O. diversifolia* | PE | Urad Zhongqi,  Nei Mongol | Chang2016024 | 2017 | 41.4011 | 109.3644 | 1546 | 249 | 2.5 | 48.2% (120) | 37.7% (94) | 14.1% (35) | 0 | 0 |
| 10 | *O. diversifolia* | GXB | Guyang County,  Nei Mongol | Chang2016018 | 2017 | 41.2838 | 109.8094 | 1560 | 82 | 0.57 | 95.1% (78) | 4.9% (4) | 0 | 0 | 0 |
| 11 | *O. diversifolia* | DT | Damao Qi,  Nei Mongol | Chang2016015 | 2017 | 41.4311 | 109.9689 | 1554 | 318 | 1.6 | 97.5% (310) | 2.5% (8) | 0 | 0 | 0 |
| 12 | *O. diversifolia* | BT | Baotou City,  Nei Mongol | Chang2016005 | 2017 | 40.7159 | 110.1027 | 1338 | 125 | 5.0 | 97.6% (122) | 2.4% (3) | 0 | 0 | 0 |
| 13 | Hybrid zone | G | Guyang County,  Nei Mongol | Chang2016016 | 2016 | 41.0492 | 109.8509 | 1524 | 82 | 3.3 | 30.5% (25) | 22.0% (18) | 14.6% (12) | 19.5% (16) | 13.4% (11) |
| 14 | Hybrid zone | HUA | Damao Qi,  Nei Mongol | Chang2017047 | 2017 | 41.7160 | 110.1717 | 1555 | 42 | 1.7 | 21.4% (9) | 26.2% (11) | 7.2% (3) | 11.9% (5) | 33.3% (14) |
| 15 | *O. leptophylla* | BOPO | Guyang County,  Nei Mongol | Chang2016011 | 2017 | 41.0899 | 110.0500 | 1418 | 33 | 0.92 | 3.0% (1) | 0 | 3.0% (1) | 9.1% (3) | 84.9% (28) |
| 16 | *O. leptophylla* | BOPT | Guyang County,  Nei Mongol | Chang2016088 | 2017 | 41.0693 | 110.1042 | 1422 | 42 | 1.7 | 0 | 0 | 0 | 0 | 100%  (42) |
| 17 | *O. leptophylla* | ZHONG | Wuchuan County, Nei Mongol | Chang2018090 | 2018 | 40.9567 | 111.5236 | 1554 | 53 | 2.1 | 0 | 0 | 0 | 0 | 100%  (53) |
| 18 | *O. leptophylla* | H | Siziwang Qi,  Nei Mongol | Chang2018105 | 2018 | 41.8767 | 111.9617 | 1500 | 25 | 0.16 | 0 | 0 | 0 | 4.0% (1) | 96.0% (24) |
| 19 | *O. leptophylla* | L | Qahar Youyi Qianqi, Nei Mongol | Chang2018120 | 2018 | 41.0958 | 113.0917 | 1400 | 26 | 0.52 | 0 | 0 | 0 | 0 | 100% (26) |

^a^All voucher specimens are deposited in the Northwest A&F University Herbarium (WUK), Yangling, Shaanxi, China.

^b^Size: estimated population size, i.e., the total number of individuals in the population.

^c^Density: estimated population density per 100 m^2^, calculated as population size divided by total area.

^d^Phenotype proportion: the proportion of each phenotype in the population, with the number of individuals for each phenotype in brackets.

**Table S2** Microsatellite loci information. All of the loci were retrieved from Wang et al. (2018). Annealing temperature was 56℃ for all loci. *N*, total number of individuals with successful amplification; *A*, total number of alleles; *N*_a_, number of alleles per population; *H*_o_, observed heterozygosity; *H*_e_, expected heterozygosity; *F*_IS_, fixation index. For those genetic diversity parameters, data are presented as mean value averaged across all 19 populations.

| **No.** | **Locus** | **Repeat motif** | **Allele size range (bp)** | **Dye**  **(group)** | ***N*** | ***A*** | ***N*_a_** | ***H*_o_** | ***H*_e_** | ***F*_IS_** |
| --- | --- | --- | --- | --- | --- | --- | --- | --- | --- | --- |
| 1 | N745892 | (ATAG)_12_ | 148–282 | 6-FAM (1) | 488 | 45 | 13.9 | 0.511 | 0.901 | 0.454 |
| 2 | N145635 | (GAG)_12_ | 87–207 | HEX (1) | 471 | 34 | 10.3 | 0.568 | 0.855 | 0.350 |
| 3 | N2724893 | (AAC)_10_ | 111–137 | ROX (1) | 492 | 10 | 5.5 | 0.497 | 0.621 | 0.198 |
| 4 | N2717495 | (TCTA)_10_ | 144–232 | HEX (2) | 467 | 18 | 8.8 | 0.316 | 0.837 | 0.616 |
| 5 | N178451 | (ATATA)_13_ | 97–183 | ROX (2) | 483 | 18 | 10.4 | 0.594 | 0.845 | 0.298 |
| 6 | N161850 | (AAT)_13_ | 102–195 | 6-FAM (3) | 484 | 30 | 15.4 | 0.735 | 0.902 | 0.175 |
| 7 | N49251 | (TCT)_11_ | 103–142 | HEX (3) | 502 | 14 | 7.6 | 0.687 | 0.764 | 0.108 |
| 8 | N350553 | (TTC)_22_ | 157–271 | HEX (3) | 472 | 39 | 9.2 | 0.356 | 0.656 | 0.466 |
| 9 | N935993 | (ATG)_10_ | 90–120 | ROX (3) | 466 | 12 | 6.5 | 0.598 | 0.679 | 0.133 |
| 10 | N2528349 | (ATCT)_20_ | 134–248 | HEX (4) | 499 | 27 | 9.8 | 0.550 | 0.727 | 0.225 |
| 11 | N2697375 | (TATG)_15_ | 136–214 | ROX (4) | 494 | 29 | 12.0 | 0.645 | 0.872 | 0.264 |

**Table S3** Primer sequences of cpDNA intergenic spacer regions amplified in this study. T_a_, annealing temperature.

| **Locus** | **Align. length (bp)** | **Primer sequence (5'-3')** | **T_a_ (°C)** | **Reference** |
| --- | --- | --- | --- | --- |
| *trn*T-*psb*D | 919 | F: CCCTTTTAACTCAGTGGTAG | 60 | Shaw *et al*. (2007) |
|  |  | R: CTCCGTARCCAGTCATCCATA |  |  |
| *pet*N-*psb*M | 863 | F: ATGGATATAGTAAGTCTYGCTTGGGC | 54 | Shaw *et al*. (2005) |
|  |  | R: ATGGAAGTAAATATTCTYGCATTTATTGCT |  |  |
| *trn*S-*trn*G | 527 | F: AACTCGTACAACGGATTAGCAATC | 53 | Shaw *et al*. (2007); |
|  |  | R: TTTTACCACTAAACTATACCCGC |  | Shaw *et al*. (2005) |
| *psb*E-*pet*L | 794 | F: TATCGAATACTGGTAATAATATCAGC | 50 | Shaw *et al*. (2007) |
|  |  | R: AGTAGAAAACCGAAATAACTAGTTA |  |  |
| *rpl*16 intron | 815 | F: GCTATGCTTAGTGTGTGACTCGTTG | 60 | Shaw *et al*. (2005) |
|  |  | R: CCCTTCATTCTTCCTCTATGTTG |  |  |
| **Total** | **3918** |  |  |  |

Shaw, J., Lickey, E. B., Beck, J. T., Farmer, S. B., Liu, W., Miller, J., Siripun, K. C., Winder, C. T., Schilling, E. E., Small, R. L. (2005). The tortoise and the hare II: relative utility of 21 noncoding chloroplast DNA sequences for phylogenetic analysis. *American Journal of Botany*, *92*, 142–166. https://doi.org/10.3732/ajb.92.1.142

Shaw, J., Lickey, E. B., Schilling, E. E., & Small, R. L. (2007). Comparison of whole chloroplast genome sequences to choose noncoding regions for phylogenetic studies in angiosperms: the tortoise and the hare III. *American Journal of Botany*, *94*, 275–288. https://doi.org/10.3732/ajb.94.3.275

**Table S4** The identification of 507 *Oxytropis* individuals based on morphological, cpDNA haplotype, and nuclear analyses (STRUCTURE, hybrid index, NEWHYBRIDS).

Table S4 is uploaded as an excel file.

**Table S5** Summary statistics of leaf-morphological traits measured from 19 *Oxytropis* populations sampled. Data are presented as mean (s.d.); the six leaf-morphological traits are in mm; PC1 and PC2 are in scaled units; trichome density on leaf upper surface is presented as the number of epidermal hairs counted on a 1mm × 1mm surface. *N*, number of individuals measured. *P*-values indicate the significance of differences among categories in linear mixed-effects models, and percentage variance explained by the random factor ‘population’ is given. Different letters indicate significant differences of pairwise comparisons (*P* < 0.05, Tukey’s HSD test).

|  | ***O. diversifolia***  ***N* = 321**  **(1, 1-3, 3 leaflets)** | **Putative hybrids^a^**  ***N* = 42**  **(1, 1-3, 3, 3-5, 5-13 leaflets)** | ***O. leptophylla***  ***N* = 102**  **(5-13 leaflets)** | ***P*-value** | **Pop.** |
| --- | --- | --- | --- | --- | --- |
| 1. **Six leaf morphological traits** | | | | | |
| Early leaf length | 6.90 (1.86) a | 6.92 (2.00) ab | 4.89 (1.58) b | 0.081 | 47.3% |
| Early leaf width | 2.09 (0.620) a | 2.00 (0.788) a | 1.16 (0.236) b | <0.05 | 36.7% |
| Mature leaf length | 13.7 (3.84) | 14.3 (4.74) | 12.2 (3.98) | 0.33 | 44.4% |
| Mature leaf width | 2.01 (0.560) a | 1.92 (0.635) a | 1.34 (0.324) b | <0.001 | 26.0% |
| Petiole length of early leaf | 8.88 (2.30) a | 8.25 (1.82) a | 11.5 (4.11) b | <0.05 | 49.4% |
| Petiole length of mature leaf | 16.0 (4.81) a | 21.3 (6.92) b | 31.3 (11.5) c | <0.001 | 42.9% |
|  |  |  |  |  |  |
| PC1 (52.3%) | 0.0640 (1.74) | -0.00550 (1.85) | -0.299 (1.72) | 0.59 | 56.3% |
| PC2 (27.1%) | -0.533 (0.858) a | -0.135 (1.02) b | 1.58 (1.11) c | <0.0001 | 25.8% |
|  |  |  |  |  |  |
| 1. **Micro-morphological characteristics of leaves** | | | | | |
| Trichome density | 21.0 (15.4) a | 3.97 (7.99) b | 0 (0) b | <0.01 | 47.3% |

^a^Putative hybrids from two populations in the hybrid zone were identified based on nuclear microsatellite data, and may include *F*_1_/*F*_2_ hybrids, backcross types and various segregation products.

**Table S6** Summary of microsatellite loci showing deviations from Hardy-Weinberg equilibrium (HWE), locus pairs in significant linkage disequilibrium (LD), and cytonuclear disequilibrium between multilocus nuclear assignment and cpDNA haplotype.

| **Population** | **No. of loci showing departure from HWE** | **No. pairs of loci in LD^a^** | **Cytonuclear** **disequilibrium^b^** |
| --- | --- | --- | --- |
| **(1) *O. diversifolia*** | | | |
| PSET | 1 | 0 | na |
| DIANO | 7 | 3(0) | na |
| HU | 8 | 6(0) | na |
| PTWE | 9 | 2(0) | na |
| PELE | 9 | 3(0) | na |
| PSIX | 9 | 1(0) | na |
| DIANT | 9 | 0 | na |
| PSEV | 8 | 2(1) | na |
| PE | 10 | 1(0) | na |
| GXB | 6 | 1(0) | na |
| DT | 7 | 1(0) | na |
| BT | 6 | 2(0) | na |
| **(2) Hybrid zone** | | | |
| G | 10 | 4(0) | *P* = 0.0072 |
| HUA | 8 | 2(0) | *P* = 0.0068 |
| **(3) *O. leptophylla*** | | | |
| BOPO | 7 | 1(0) | na |
| BOPT | 5 | 0 | na |
| ZHONG | 8 | 3(0) | na |
| H | 6 | 0 | na |
| L | 5 | 1(0) | na |

^a^The number of loci remaining significant after Benjamini–Hochberg correction is shown in parentheses.

^b^Cytonuclear disequilibrium is tested in hybrid zone localities G and HUA. cpDNA haplotypes were set as biallelic (*O. diversifolia* haplotype and *O. leptophylla* haplotype), and multilocus nuclear assignments were used (*O. diversifolia*, putative hybrids, *O. leptophylla*).

**Table S7** Estimates of multi-locus genetic diversity based on nuclear microsatellite dataset for 19 populations sampled. *N*_1_, number of 1-leaflet individuals genotyped; *N*_2_, number of 1–3-leaflet individuals genotyped; *N*_3_, number of 3-leaflet individuals genotyped; *N*_4_, number of 3–5-leaflet individuals genotyped; *N*_5_, number of 5–13-leaflet individuals genotyped; *N*_na_, number of individuals genotyped with unknown leaf-shape phenotype; *N*, total number of individuals genotyped. *N*_a_, number of alleles; *H*_o_, observed heterozygosity; *H*_e_, unbiased expected heterozygosity; *F*_IS_, fixation index. For those genetic diversity parameters, data are presented as mean value averaged across 11 loci.

| **Population** | ***N*_1_** | ***N*_2_** | ***N*_3_** | ***N*_4_** | ***N*_5_** | ***N*_na_** | ***N*** | ***N*_a_** | ***H*_o_** | ***H*_e_** | ***F*_IS_** |
| --- | --- | --- | --- | --- | --- | --- | --- | --- | --- | --- | --- |
| **(1) *O. diversifolia*** | **116** | **99** | **98** | **2** | **0** | **6** | **321** | **22.7** | **0.583** | **0.845** | **0.307** |
| PSET | 0 | 1 | 0 | 0 | 0 | 6 | 7 | 5.4 | 0.572 | 0.772 | 0.273 |
| DIANO | 3 | 6 | 22 | 1 | 0 | 0 | 32 | 11.5 | 0.575 | 0.805 | 0.286 |
| HU | 4 | 21 | 7 | 0 | 0 | 0 | 32 | 10.9 | 0.585 | 0.818 | 0.290 |
| PTWE | 4 | 11 | 17 | 0 | 0 | 0 | 32 | 12.5 | 0.585 | 0.824 | 0.290 |
| PELE | 14 | 3 | 13 | 0 | 0 | 0 | 30 | 11.2 | 0.555 | 0.824 | 0.327 |
| PSIX | 4 | 14 | 14 | 0 | 0 | 0 | 32 | 12.5 | 0.614 | 0.841 | 0.273 |
| DIANT | 10 | 13 | 10 | 0 | 0 | 0 | 33 | 12.4 | 0.599 | 0.836 | 0.282 |
| PSEV | 11 | 12 | 9 | 0 | 0 | 0 | 32 | 12.1 | 0.644 | 0.840 | 0.233 |
| PE | 13 | 9 | 6 | 1 | 0 | 0 | 29 | 12.9 | 0.561 | 0.843 | 0.336 |
| GXB | 18 | 2 | 0 | 0 | 0 | 0 | 20 | 10.4 | 0.579 | 0.827 | 0.301 |
| DT | 17 | 5 | 0 | 0 | 0 | 0 | 22 | 10.3 | 0.562 | 0.831 | 0.312 |
| BT | 18 | 2 | 0 | 0 | 0 | 0 | 20 | 7.4 | 0.509 | 0.795 | 0.350 |
| **(2) Hybrid zone** | **23** | **16** | **5** | **6** | **30** | **4** | **84** | **15.0** | **0.526** | **0.795** | **0.335** |
| G | 19 | 10 | 1 | 3 | 20 | 2 | 55 | 12.9 | 0.533 | 0.801 | 0.332 |
| HUA | 4 | 7 | 4 | 3 | 11 | 0 | 29 | 10.0 | 0.513 | 0.758 | 0.322 |
| **(3) *O. leptophylla*** | **0** | **1** | **0** | **5** | **96** | **0** | **102** | **13.1** | **0.495** | **0.729** | **0.319** |
| BOPO | 0 | 1 | 0 | 1 | 18 | 0 | 20 | 7.0 | 0.460 | 0.669 | 0.308 |
| BOPT | 0 | 0 | 0 | 1 | 18 | 0 | 19 | 8.0 | 0.548 | 0.715 | 0.241 |
| ZHONG | 0 | 0 | 0 | 0 | 22 | 0 | 22 | 7.9 | 0.487 | 0.729 | 0.333 |
| H | 0 | 0 | 0 | 2 | 19 | 0 | 21 | 6.6 | 0.461 | 0.697 | 0.344 |
| L | 0 | 0 | 0 | 1 | 19 | 0 | 20 | 7.1 | 0.517 | 0.730 | 0.295 |
| **Total** | **139** | **116** | **103** | **13** | **126** | **10** | **507** | **25.1** | **0.558** | **0.843** | **0.336** |

**Table S8** Summary of nucleotide diversity for cpDNA dataset. *N*_1_, number of 1-leaflet individuals sequenced; *N*_2_, number of 1–3-leaflet individuals sequenced; *N*_3_, number of 3-leaflets individuals sequenced; *N*_4_, number of 3–5-leaflet individuals sequenced; *N*_5_, number of 5–13-leaflet individuals sequenced; *N*_na_, number of individuals genotyped with unknown leaf-shape phenotype; *N*, total number of individuals sequenced. *S*, number of segregating sites; *h*, number of haplotypes; *h*_p_, number of private haplotypes (i.e., haplotypes that occur in only one population); *Hd*, haplotype diversity; *π*, nucleotide diversity. ns, not significant, *P* > 0.10; + *P* > 0.05; * *P* < 0.05; ** *P* < 0.01.

| **Population** | ***N*_1_** | ***N*_2_** | ***N*_3_** | ***N*_4_** | ***N*_5_** | ***N*_na_** | ***N*** | ***S*** | ***h*** | ***h*_p_** | ***Hd*** | ***π*** | **Tajima’s *D*** | **Fu and Li’s *D****** | **Fu and Li’s *F****** |
| --- | --- | --- | --- | --- | --- | --- | --- | --- | --- | --- | --- | --- | --- | --- | --- |
| **(1) *O. diversifolia*** | **55** | **47** | **45** | **1** | **0** | **6** | **154** | **54** | **51** | **37** | **0.910** | **0.00063** | **-2.339**** | **-5.798**** | **-5.187**** |
| PSET | 0 | 0 | 0 | 0 | 0 | 6 | 6 | 6 | 5 | 2 | 0.933 | 0.00067 | -0.496ns | -0.416ns | -0.463ns |
| DIANO | 3 | 4 | 7 | 1 | 0 | 0 | 15 | 8 | 8 | 4 | 0.790 | 0.00049 | -1.060ns | -1.737ns | -1.781ns |
| HU | 4 | 6 | 5 | 0 | 0 | 0 | 15 | 12 | 10 | 6 | 0.924 | 0.00075 | -1.141ns | -1.282ns | -1.428ns |
| PTWE | 4 | 6 | 5 | 0 | 0 | 0 | 15 | 10 | 11 | 4 | 0.952 | 0.00061 | -1.119ns | -1.595ns | -1.681ns |
| PELE | 6 | 3 | 6 | 0 | 0 | 0 | 15 | 7 | 7 | 3 | 0.838 | 0.00051 | -0.542ns | -0.972ns | -0.981ns |
| PSIX | 4 | 6 | 7 | 0 | 0 | 0 | 17 | 8 | 8 | 1 | 0.838 | 0.00046 | -1.075ns | -0.799ns | -1.008ns |
| DIANT | 5 | 5 | 5 | 0 | 0 | 0 | 15 | 10 | 9 | 3 | 0.886 | 0.00063 | -1.001ns | -0.734ns | -0.925ns |
| PSEV | 5 | 5 | 5 | 0 | 0 | 0 | 15 | 7 | 7 | 1 | 0.819 | 0.00040 | -1.145ns | -1.220ns | -1.375ns |
| PE | 6 | 4 | 5 | 0 | 0 | 0 | 15 | 15 | 9 | 4 | 0.905 | 0.00093 | -1.172ns | -1.363ns | -1.506ns |
| GXB | 6 | 2 | 0 | 0 | 0 | 0 | 8 | 9 | 6 | 2 | 0.929 | 0.00071 | -1.375ns | -1.566ns | -1.686ns |
| DT | 6 | 4 | 0 | 0 | 0 | 0 | 10 | 14 | 10 | 5 | 1.000 | 0.00092 | -1.610+ | -1.878+ | -2.041+ |
| BT | 6 | 2 | 0 | 0 | 0 | 0 | 8 | 3 | 4 | 2 | 0.750 | 0.00026 | -0.525ns | -0.176ns | -0.282ns |
| **(2) Hybrid zone** | **14** | **12** | **5** | **6** | **26** | **1** | **64** | **41** | **15** | **2** | **0.812** | **0.00391** | **2.118*** | **0.860ns** | **1.593ns** |
| G | 11 | 8 | 1 | 3 | 19 | 1 | 43 | 38 | 12 | 2 | 0.804 | 0.00387 | 2.096* | 0.829ns | 1.512ns |
| HUA | 3 | 4 | 4 | 3 | 7 | 0 | 21 | 33 | 8 | 0 | 0.762 | 0.00401 | 2.316* | 0.982ns | 1.614* |
| **(3) *O. leptophylla*** | **0** | **1** | **0** | **4** | **38** | **0** | **43** | **21** | **4** | **2** | **0.550** | **0.00062** | **-1.779ns** | **1.008ns** | **0.074ns** |
| BOPO | 0 | 1 | 0 | 1 | 6 | 0 | 8 | 1 | 2 | 0 | 0.571 | 0.00016 | 1.444ns | 0.888ns | 1.100ns |
| BOPT | 0 | 0 | 0 | 1 | 7 | 0 | 8 | 1 | 2 | 0 | 0.571 | 0.00016 | 1.167ns | 0.888ns | 1.032ns |
| ZHONG | 0 | 0 | 0 | 0 | 9 | 0 | 9 | 21 | 4 | 2 | 0.694 | 0.00218 | 0.068ns | 1.210ns | 1.047ns |
| H | 0 | 0 | 0 | 2 | 7 | 0 | 9 | 1 | 2 | 0 | 0.389 | 0.00011 | 0.156ns | 0.840ns | 0.748ns |
| L | 0 | 0 | 0 | 0 | 9 | 0 | 9 | 1 | 2 | 0 | 0.389 | 0.00011 | 0.156ns | 0.840ns | 0.748ns |
| **Total** | **69** | **60** | **50** | **11** | **64** | **7** | **261** | **80** | **57** | **41** | **0.913** | **0.00341** | **-0.317ns** | **-4.635**** | **-3.115**** |

**Table S9** Summary statistics for microhabitat variables estimated from 18 *Oxytropis* populations sampled. Data are presented as mean (s.d.). *N*, number of individuals. *P*-values indicate the significance of differences among categories in linear mixed-effects models, and percentage variance explained by the random factor ‘population’ is given. Different letters indicate significant differences of pairwise comparisons (*P* < 0.05, Tukey’s HSD test).

|  | ***O. diversifolia***  ***N* = 314**  **(1, 1-3, 3 leaflets)** | **Hybrid zone**  ***N* = 62**  **(1, 1-3, 3, 3-5, 5-13 leaflets)** | ***O. leptophylla***  ***N* = 101**  **(5-13 leaflets)** | ***P*-value** | **Pop.** |
| --- | --- | --- | --- | --- | --- |
| **Slope (°)** | **4.95 (3.63) a** | **10.1 (6.41) b** | **12.9 (9.74) c** | **<0.01** | **44.0%** |
| Rocky ground (%) | 22.2 (18.9) | 41.2 (19.1) | 27.0 (16.9) | 0.15 | 45.0% |
| Vegetation cover (%) | 28.8 (18.7) | 24.4 (13.6) | 32.8 (15.0) | 0.69 | 52.0% |
| Bare ground (%) | 49.0 (21.9) | 34.5 (12.6) | 40.1 (16.3) | 0.48 | 57.0% |

**Table S10** Bioclimatic variables (named BIO1 to BIO19) downloaded from WorldClim for the macroclimatic-association analysis. Variables marked with an asterisk (*) were used for the PCA of 54 localities of two parental species and hybrid zones. Data are presented as mean (s.d.). *P*-values indicate the significance of differences among categories in one-way ANOVA. Different letters indicate significant differences of pairwise comparisons (*P* < 0.05, Tukey’s HSD test). Cor. represents the highly correlated bioclimatic variable with Pearson correlation coefficients (*r*) also shown. *** *P*< 0.001.

| **Code** | **Variable** | ***O. diversifolia***  **(12 localities)** | **Hybrid zone**  **(2 localities)** | ***O. leptophylla***  **(40 localities)** | ***P*-value** | **Cor.** | ***r*** |
| --- | --- | --- | --- | --- | --- | --- | --- |
| BIO1 | Annual Mean Temperature (°C) | 4.75 (0.673) | 4.34 (0.498) | 3.22 (2.28) | 0.07 |  |  |
| BIO2 | Mean Diurnal Range (Mean of monthly (max temp - min temp)) (°C) | 13.2 (0.544) | 13.3 (0.0766) | 13.0 (1.01) | 0.85 |  |  |
| BIO3 | Isothermality (BIO2/BIO7) (*100) (%) | 27.1 (0.609) | 27.8 (0.201) | 26.5 (1.80) | 0.35 |  |  |
| BIO4 | Temperature Seasonality (standard deviation *100) (°C) | 1290 (23.6) | 1260 (4.40) | 1331 (141) | 0.47 |  |  |
| **BIO5*** | **Max Temperature of Warmest Month (°C)** | **27.7 (0.584) a** | **26.9 (0.212) ab** | **26.3 (1.36) b** | **<0.01** |  |  |
| BIO6 | Min Temperature of Coldest Month (°C) | -21.0 (1.22) | -21 (0.141) | -23.1 (4.52) | 0.26 |  |  |
| BIO7 | Temperature Annual Range (BIO5-BIO6) (°C) | 48.7 (1.20) | 47.9 (0.707) | 49.4 (4.36) | 0.76 |  |  |
| BIO8 | Mean Temperature of Wettest Quarter (°C) | 18.4 (0.796) | 17.4 (0.424) | 18.5 (1.32) | 0.45 |  |  |
| BIO9 | Mean Temperature of Driest Quarter (°C) | -11.6 (0.849) | -11.9 (0.318) | -13.6 (3.48) | 0.12 |  |  |
| **BIO10** | **Mean Temperature of Warmest Quarter (°C)** | **20.0 (0.611) a** | **19.2 (0.401) ab** | **18.8 (1.42) b** | **<0.05** | **bio05** | **0.93****9***** |
| BIO11 | Mean Temperature of Coldest Quarter (°C) | -11.9 (0.836) | -11.9 (0.318) | -14.0 (3.92) | 0.14 |  |  |
| **BIO12*** | **Annual Precipitation (mm)** | **230 (53.3) a** | **272 (34.6) ab** | **356 (68.8) b** | **<0.0001** |  |  |
| **BIO13** | **Precipitation of Wettest Month (mm)** | **64.2 (16.0) a** | **76.5 (10.6) ab** | **107 (25.1) b** | **<0.0001** | **bio12** | **0.965***** |
| BIO14 | Precipitation of Driest Month (mm) | 2.42 (0.996) | 3 (1.41) | 2.17 (0.931) | 0.41 |  |  |
| **BIO15*** | **Precipitation Seasonality (Coefficient of Variation) (%)** | **108 (3.98) a** | **111 (4.58) ab** | **114 (6.92) b** | **<0.05** |  |  |
| **BIO16** | **Precipitation of Wettest Quarter (mm)** | **152 (33.9) a** | **180 (17.7) ab** | **245 (50.0) b** | **<0.0001** | **bio12** | **0.981***** |
| BIO17 | Precipitation of Driest Quarter (mm) | 8.58 (3.20) | 10.5 (4.95) | 8.35 (3.10) | 0.64 |  |  |
| **BIO18** | **Precipitation of Warmest Quarter (mm)** | **150 (33.6) a** | **179 (17.0) ab** | **245 (49.7) b** | **<0.0001** | **bio12** | **0.979***** |
| BIO19 | Precipitation of Coldest Quarter (mm) | 8.58 (3.20) | 10.5 (4.95) | 8.43 (3.14) | 0.67 |  |  |

**Table S11** The occurrence of associated plant species in 19 *Oxytropis* localities we surveyed.

Table S11 is uploaded as an excel file together with Table S4.

**Table S12** Summary of geographic cline analysis. (A) The AICc (the Akaike Information Criterion score corrected for small sample size) values and Log-likelihood scores of fitted clines under different models using the ‘HZAR’ package in R. (B) Parameter estimates for the model that best fits the data. Two log-likelihood unit support limits are presented in parentheses. *c*, cline center measured in distance (km) from the center of the collapsed 1-D transect; *w*, cline width presented as 1 / maximum slope; *P*_min_ / *μ*_L_, the minimum estimated allele frequency / quantitative trait mean at the west end of the cline; *P*_max_ / *μ*_R_, the maximum estimated allele frequency / quantitative trait mean at the eastern end; δ and τ are shape parameters for the exponential decay curves (tails).

|  | **Leaf-shape phenotype^a^** | **Leaf morphology^b^**  **(PC2 scores)** | **cpDNA**  **haplotype^a^** | **Microsatellite genotype^b^**  **(*Q_K2_* scores)** | **Microhabitat^b^**  **In(slope)** |
| --- | --- | --- | --- | --- | --- |
| **(A) Model selection: AICc (In *L*)** | | | | | |
| Model I (parameters = 2) | 88.1 (-42.1) | 1188.5 (-591.2) | 9.1* (-2.5) | -902.8 (454.4) | 873.8 (-433.9) |
| Model II (parameters = 4) | 49.7* (-20.9) | 1158.8* (-572.2) | 13.4 (-2.6) | -1004.4 (509.3) | 789.2* (-387.4) |
| Model III (parameters = 8) | 1097.0 (-540.4) | 1163.1 (-570.2) | 21.7 (-2.6) | -1057.2* (539.9) | 797.3 (-387.3) |
| **(B) Parameter estimates** | | | | | |
| *c* | 0.69 (-1.6~2.9) | 1.4 (-4.7~9.5) | -8.2 (-10.0~-6.3) | -8.3 (-8.3~-7.5) | -16.6 (-24.1~-9.2) |
| *w* | 17.2 (13.8~20.5) | 29.2 (8.6~48.8) | 13.5 (8.7~22.5) | 6.3 (6.1~7.6) | 53.7 (16.0~80.4) |
| *P*_min_ / *μ*_L_ | 0 (0~0.0015) | -0.531 (-0.629~-0.396) | 0 (fixed) | 0.031 (0.030~0.040) | 1.229 (1.146~1.306) |
| *P*_max_ / *μ*_R_ | 0.993 (0.968~0.999) | 1.704 (1.460~2.033) | 1 (fixed) | 0.988 (0.985~0.990) | 2.357 (2.196 ~2.507) |
| *δ*_L_ | no tail | no tail | no tail | 23.9 (18.2~43.0) | no tail |
| *τ*_L_ | no tail | no tail | no tail | 0.050 (0.016~0.262) | no tail |
| *δ*_R_ | no tail | no tail | no tail | 9.5 (9.5~11.8) | no tail |
| *τ*_R_ | no tail | no tail | no tail | 0.041 (0.032~0.056) | no tail |

* An asterisk denotes the model that provide the best fit with the fewest number of estimated parameters.

^a^analyzed as allele frequency data

^b^analyzed as quantitative trait data
